# Hypoxia-inducible factor upregulation by roxadustat attenuates drug reward by altering brain iron homoeostasis

**DOI:** 10.1038/s41392-023-01578-2

**Published:** 2023-09-18

**Authors:** Pengju Yan, Ningning Li, Ming Ma, Zhaoli Liu, Huicui Yang, Jinnan Li, Chunlei Wan, Shuliu Gao, Shuai Li, Longtai Zheng, John L. Waddington, Lin Xu, Xuechu Zhen

**Affiliations:** 1 Jiangsu Key Laboratory of Neuropsychiatric Diseases and College of Pharmaceutical Sciences, Soochow University, Suzhou, 215123 China; 2 CAS Key Laboratory of Animal Models and Human Disease Mechanisms, and KIZ-SU Joint Laboratory of Animal Model and Drug Development, and Laboratory of Learning and Memory, Kunming Institute of Zoology, the Chinese Academy of Sciences, Kunming, 650223 China; 3 School of Pharmacy and Biomolecular Sciences, RCSI University of Medicine and Health Sciences, Dublin 2, Ireland

**Keywords:** Molecular neuroscience, Molecular medicine

## Abstract

Substance use disorder remains a major challenge, with an enduring need to identify and evaluate new, translational targets for effective treatment. Here, we report the upregulation of Hypoxia-inducible factor-1α (HIF-1α) expression by roxadustat (Rox), a drug developed for renal anemia that inhibits HIF prolyl hydroxylase to prevent degradation of HIF-1α, administered either systemically or locally into selected brain regions, suppressed morphine (Mor)-induced conditioned place preference (CPP). A similar effect was observed with methamphetamine (METH). Moreover, Rox also inhibited the expression of both established and reinstated Mor-CPP and promoted the extinction of Mor-CPP. Additionally, the elevation of HIF-1α enhanced hepcidin/ferroportin 1 (FPN1)-mediated iron efflux and resulted in cellular iron deficiency, which led to the functional accumulation of the dopamine transporter (DAT) in plasma membranes due to iron deficiency-impaired ubiquitin degradation. Notably, iron-deficient mice generated via a low iron diet mimicked the effect of Rox on the prevention of Mor- or METH-CPP formation, without affecting other types of memory. These data reveal a novel mechanism for HIF-1α and iron involvement in substance use disorder, which may represent a potential novel therapeutic strategy for the treatment of drug abuse. The findings also repurpose Rox by suggesting a potential new indication for the treatment of substance use disorder.

## Introduction

Substance use disorder is a mental disorder characterized by compulsive drug seeking and marked symptoms of dependence.^1–3^ Chronic exposure to addictive drugs such as methamphetamine (METH) and morphine (Mor) leads to the activation of reward circuits from the ventral tegmental area (VTA) to the nucleus accumbens (NAc), the prefrontal cortex (PFC) and the striatum (STR). This increase in dopamine activity is considered as a common basis for the development of reward-related behaviors. METH can competitively bind to the dopamine transporter (DAT) to inhibit the uptake of dopamine from the synapse; it also promotes internalization of DAT from the plasma membrane,^4,5^ which further reduces the uptake activity of DAT. In contrast, morphine acts to reduce inhibition from GABAergic projections onto dopaminergic neurons and facilitate dopamine release.^6–8^ Regardless of the drug, substance-induced activation of dopaminergic activity plays a prominent role in the development of drug reward.

Hypoxia-inducible factor-1α (HIF-1α) is a key transcriptional regulator of oxidative stress and is considered a sensor for hypoxia.^9,10^ HIF-1α has been shown to regulate cell survival and a wide spectrum of cellular functions.^11,12^ Administration of addictive drugs such as amphetamines and opioids can alter HIF-1α and downstream gene expression in vivo and in vitro.^13–20^ Interestingly, HIF-1α is reported to be a critical modulator in iron metabolism^21,22^ and upregulation of HIF-1α resulted in decreased cellular iron accumulation.^23,24^ Moreover, iron homeostasis is involved in the regulation of dopamine concentration due to its role in dopamine synthesis and monoamine oxidase activity.^25–27^ Mounting evidence indicates dysregulation of iron homeostasis in substance use disorder in both humans and animal models.^28–31^

Recent data indicate that iron homeostasis plays an important role in DAT expression through PKC-mediated ubiquitination-dependent degradation. Moreover, our previous study found that mitochondrial coq7 (mClk1) deficiency-induced upregulation of HIF-1α which resulted in cellular iron deficiency and DAT membrane accumulation, ultimately, lead to attenuation of METH-induced conditioned place preference (CPP),^32^ which further implicating a critical role for HIF-1α-mediated iron metabolism in the development of drug reward.

The present work was designed to investigate the effects of modulating HIF-1α expression on morphine-induced drug reward by employing roxadustat (Rox)/FG-4592, a marketed HIF-PHD inhibitor for renal anemia. We find that, in addition to suppression of METH-CPP formation, Rox administration also inhibited Mor-induced CPP expression and reinstatement, and promoted CPP extinction. Mechanistically, these effects of Rox on drug reward paradigms were associated with increases in functional DAT expression that resulted from impaired DAT ubiquitin, which was attributed to the upregulation of HIF-1α-mediated iron export activity due to the reduced production of hepcidin. We further demonstrate that lowered iron concentrations, systemically or locally in selected brain regions, attenuates the development of psychostimulant-mediated CPP acquisition, revealing a critical role for cellular iron content in drug reward. The present studies indicate a novel and important role for iron homeostasis in drug reward effects. Moreover, they indicate a novel treatment strategy for substance use disorders through targeting HIF-1α or other regulators of iron metabolism.

## Results

### Upregulation of HIF-1α expression by roxadustat (Rox) selectively suppresses drug rewarding behaviors

We first verified the action of Rox to upregulate HIF-1α expression in vitro and in vivo in our system. Rox treatment upregulated HIF-1α expression in a dose- and time-dependent manner in PC12 cells without affecting cell viability (Supplemental Fig. 1a, b). The functional activity of Rox was monitored by measuring HIF-1α nuclear translocation and transcriptional activity on VEGF mRNA expression in PC12 cells (Supplemental Fig. 1c-f). Administration of Rox also induced upregulation of HIF-1α in the brain of mice (Supplemental Fig. 1g). We found that a 6-hour treatment with 10.0 mg/kg Rox manifested robust effects on HIF-1α expression in vivo and thus selected these treatment parameters to conduct subsequent experiments.

**Fig. 1:**
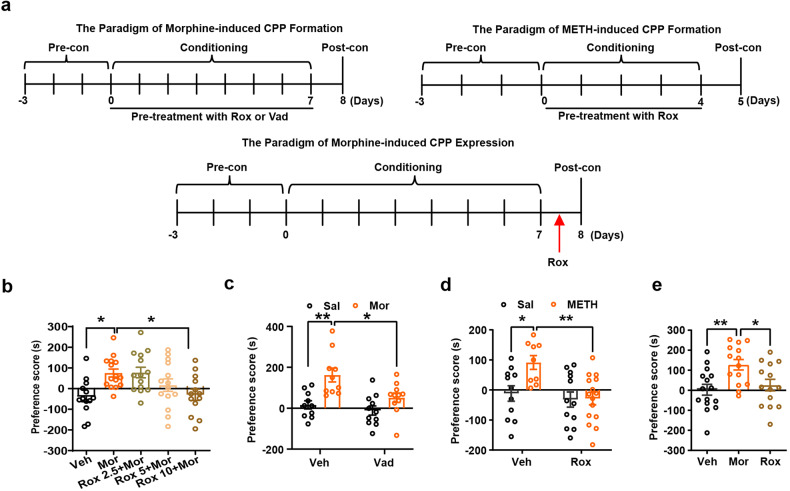
Roxaduatat (Rox) inhibits morphine (Mor)- and methamphetamine (METH)- induced CPP acquisition and expression. **a** The paradigm of morphine- and METH-induced CPP formation and expression. In the CPP acquisition test, the mouse was treated with Rox or Vad in the conditioning phase; In the CPP expression test, mouse received a single injection of Rox 6 h before postconditioning test. **b** 6 h pretreatment with Rox inhibited Mor-CPP acquisition as indicated by reduction in CPP scores as compared with Mor group (Veh, *n* = 13; Mor, *n* = 15; Rox 2.5+Mor, *n* = 14; Rox 5+Mor, *n* = 14; Rox 10+Mor, *n* = 13; group, F(4,64) = 4.790, P = 0.0019). **c** 6 h pretreatment with Vadadustat (Vad, 10 mg/kg) inhibited Mor-CPP acquisition as indicated by reduction in CPP scores (Veh-Sal, *n* = 10; Veh-Mor, *n* = 10; Vad+Sal, *n* = 11; Vad+Mor, *n* = 11; group, *F*_(1,38)_ = 16.92, *P* = 0.0002; drug, *F*_(1,38)_ = 7.483, *P* = 0.0094; group × drug, *F*_(1,38)_ = 2.701, *P* = 0.1085). **d** 6 h pretreatment with Rox (10 mg/kg) inhibited METH-CPP acquisition as indicated by reduction in CPP scores (Veh-Sal, *n* = 11; Veh-METH, *n* = 9; Rox+Sal, *n* = 12; Rox+METH, *n* = 15; group, *F*_(1,43)_ = 4.867, *P* = 0.0328; drug, *F*_(1,43)_ = 8.431, *P* = 0.0058; group × drug, *F*_(1,43)_ = 4.279, *P* = 0.0446). **e** A single injection of Rox (10 mg/kg, 6 h pretreatment) inhibited Mor-CPP expression as indicated by reduction in CPP scores (Veh, *n* = 16; Mor, *n* = 15; Rox, *n* = 15; group, F_(2,40)_ = 5.808, *P* = 0.0061). Values are presented as means ± SEM. Statistical analyses for **b**, **e** and **c**, **d** were performed using one-way ANOVA and two-way ANOVA followed by Bonferroni-corrected tests, respectively. ^*^P < 0.05, ^**^P < 0.01^.^ Veh: vehicle; Sal: saline

Mor-CPP was conducted as outlined in Fig. 1a schematic diagram for morphine CPP. We found that a 6-hour pretreatment with Rox before Mor in the conditioning phase attenuated the development of CPP in a dose-dependent manner (Fig. 1b), indicating that this drug indeed suppressed Mor-CPP acquisition. To determine if this effect is specific for Rox, vadadustat, another HIF-α hydroxylase inhibitor, was employed. As expected, vadadustat (10.0 mg/kg) also attenuated Mor-CPP acquisition (Fig. 1c). Moreover, the effect of Rox on CPP formation was not limited to Mor; this treatment also inhibited the acquisition of METH-induced CPP (Fig. 1a schematic diagram for METH and Fig. 1d). To explore if Rox administration also inhibits the expression of established Mor-CPP behavior, a single Rox treatment before CPP testing also inhibited the face of an established Mor-CPP (Fig. 1a schematic diagram for CPP expression and Fig. 1d).

Activation of transcriptional factors △fosB and CREB are considered endogenous biomarkers for addictive drug-induced CPP development.^33,34^ Although upregulation of HIF-1α by Rox alone did not alter basal fluorescence intensity of △fosB staining in STR, NAc and PFC, treatment with Rox markedly inhibited Mor-enhanced expression as evidenced by the number and fluorescence intensity of △fosB immunoreactive cells in all brain regions examined (Supplemental Fig. 2a) and Mor- and METH-induced phosphorylation of CREB (S133) in STR while attenuating CPP formation (Supplemental Fig. 2b, c).

**Fig. 2:**
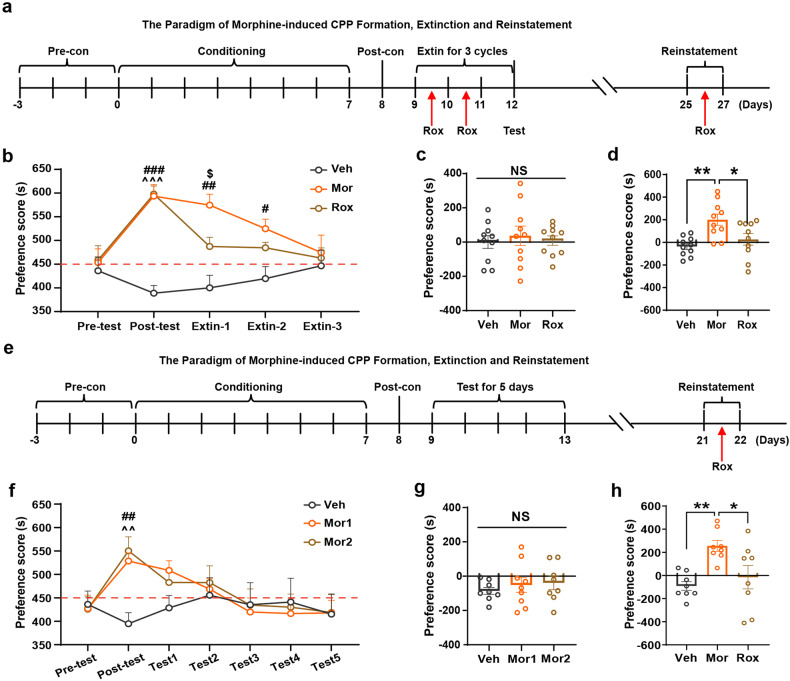
Rox promotes Mor-CPP extinction and inhibits Mor-CPP reinstatement. **a** The paradigm of Mor-CPP formation, extinction and reinstatement. As described in Methods section (*Pattern 1*), the CPP established mouse was trained to extinct, Rox was administrated instate of morphine after postconditioning phase for 3 cycles and a single Rox injection was conducted 6 h before reinstatement test. **b** Time spent in drug-paired box in Mor-CPP formation and extinction periods. Rox (10 mg/kg) promoted extinction of Mor-CPP as indicated by faster reduction in CPP scores (Veh, Mor and Rox, *n* = 10; group, *F*_(2,135)_ = 24.26, *P* < 0.0001; period, *F*_(4,135)_ = 4.217, *P* = 0.0030; group × period, *F*_(8,135)_ = 3.894, *P* = 0.0004). **c** Saline injection did not induce reinstatement of Mor-CPP (Veh, Mor and Rox, *n* = 10; F_(2,27)_ = 0.1959, *P* = 0.8232). **d** Mor (5 mg/kg) triggered Mor-CPP reinstatement, whereas it was inhibited by Rox (10 mg/kg) treatment (Veh, Mor and Rox, *n* = 10; F_(2,27)_ = 7.725, *P* = 0.0022). **e** The paradigm of Mor-CPP formation, extinction and reinstatement. As described in Methods section (*Pattern 2*), the mouse, CPP well established, was trained to extinct by repeat preference tests for 5 consecutive days, Rox was administrated instate of morphine after postconditioning phase for 3 cycles and a single Rox injection was conducted 6 h before reinstatement test. **f** Mor group was divided into Mor1 and Mor2 groups. Time spent in Mor-paired box in CPP formation and extinction periods (Veh, *n* = 12; Mor1, *n* = 14; Mor2, *n* = 12; group, *F*_(2,245)_ = 1.862, *P* = 0.1576; period, *F*_(6,245)_ = 2.431, *P* = 0.0266; group × period, *F*_(12,245)_ = 1.173, *P* = 0.3030). **g** Saline injection did not induce reinstatement of Mor-CPP (Veh, *n* = 8; Mor1, *n* = 9; Mor2, *n* = 8; group, *F*_(2,22)_ = 0.4210, *P* = 0.6616). **h** Mor (5 mg/kg) successfully triggered Mor-CPP reinstatement, whereas it was inhibited by Rox (10 mg/kg) treatment (Veh, Mor and Rox, *n* = 8; F_(2,21)_ = 7.110, *P* = 0.0044). Values are presented as means ± SEM. Statistical analyses for **b**, **f** and and for **c**, **d**, **g** and **h** were performed using two-way ANOVA and one-way ANOVA followed by Bonferroni-corrected tests, respectively. ^*^P < 0.05, ^**^P < 0.01; ^#^*P* < 0.05, ^##^*P* < 0.01, ^###^*P* < 0.001 Mor or Mor1 vs Veh; ^^^^*P* < 0.01, ^^^^^*P* < 0.001 Rox or Mor2 vs. Veh; ^$^*P* < 0.05 Mor vs Rox^;^ NS: not significant. Veh: vehicle; Sal: saline

A high relapse rate is one of the greatest challenges in drug abuse therapy, which may be attributed in part to abnormal memory.^35,36^ We next employed a confined CPP extinction paradigm (*Pattern 1* in Methods) to investigate whether upregulation of HIF-1α by Rox treatment affects drug-related memory extinction and retrieval (Fig. 2a). Results showed that daily injection of Rox accelerated extinction of established Mor-CPP. Rox-treated mice showed faster extinction relative to those receiving saline treatment (Fig. 2b). We further explored the effect of Rox on memory retrieval of CPP. As expected, saline pretreatment did not induce place preference memory retrieval (Fig. 2c), whereas Rox pretreatment markedly attenuated Mor (5.0 mg/kg)-reinstated CPP relative to controls (Fig. 2d). To further confirm this observation, we applied another extinction model to test the inhibition efficacy of Rox on CPP reinstatement (*Pattern 2* in Methods, Fig. 2e). Mice with established Mor-CPP were reintroduced into a free access CPP extinction paradigm for 5 consecutive days of exposure to the conditioning context in order to eliminate Mor-related memory. The results further confirmed that Rox administration inhibited CPP reinstatement (Fig. 2f-h).

In order to exclude any potential effects of Rox treatment on basal locomotor activity and other behaviors that may disrupt CPP formation, we also conducted a series of behavioral tests following Rox treatment. As shown in Supplemental Fig. 3a-d, Supplemental Fig. 4a-i and Supplemental Fig. 5a-i, Rox treatment did not affect locomotor activity, anxiety-like behavior or basic learning and memory processes.

### Local modulation HIF-1α expression in respective brain regions alters Mor-CPP

**Fig. 3:**
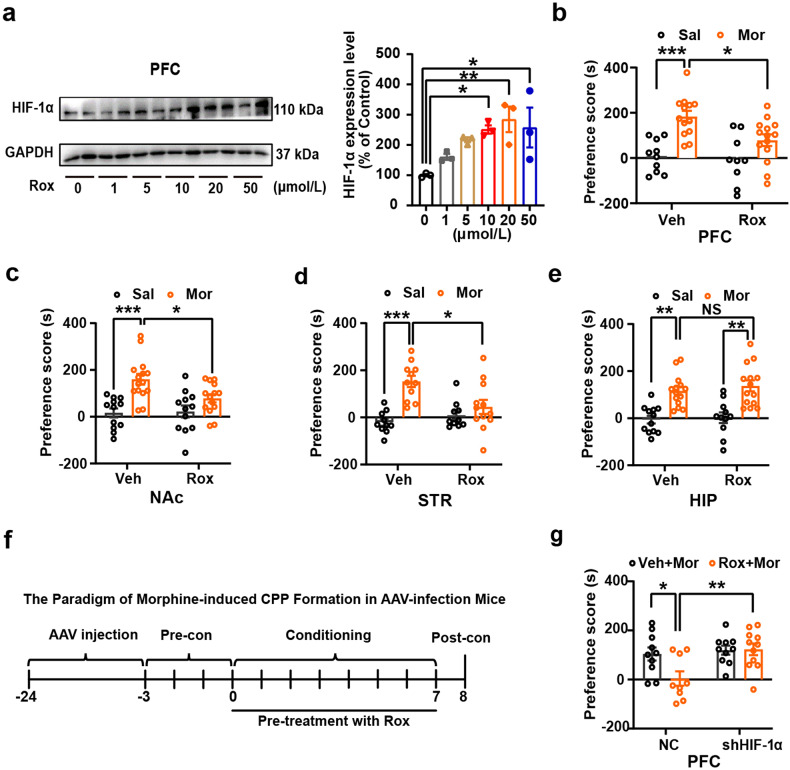
Local infusion of Rox selectively attenuates Mor-CPP acquisition and knockdown of HIF-1α abolishes Rox efficiency on Mor-CPP. Bilateral cannulae was implanted into PFC, NAc (shell), STR and HIP 7 days prior to CPP performance, respectively. **a** Local infusion of Rox (20 μmol/L, 2 μL per site) for 2 h dose-dependently upregulated HIF-1α expression in PFC (*n* = 3, *F*_(5,12)_ = 4.451, *P* = 0.0159). **b**–**e** Local infusion of Rox 2 h before Mor injection every day in conditioning phase inhibited Mor-CPP acquisition in following brain regions: PFC (Veh-Sal, *n* = 10; Veh-Mor, *n* = 13; Rox-Sal, *n* = 9; Rox-Mor, *n* = 14; group, *F*_(1,42)_ = 21.97, *P* < 0.0001; drug, *F*_(1,42)_ = 4.694, *P* = 0.0360; group × drug, *F*_(1,42)_ = 2.631, *P* = 0.1122); NAc (shell) (Veh-Sal, *n* = 12; Veh-Mor, *n* = 15; Rox-Sal, *n* = 12; Rox-Mor, *n* = 14; group, *F*_(1,49)_ = 21.05, *P* < 0.0001; drug, *F*_(1,49)_ = 2.981, *P* = 0.0905; group × drug, *F*_(1,49)_ = 4.043, *P* = 0.0499); and STR (Veh-Sal, *n* = 10; Veh-Mor, *n* = 11; Rox-Sal, *n* = 10; Rox-Mor, *n* = 12; group, *F*_(1,39)_ = 19.69, *P* < 0.0001; drug, *F*_(1,39)_ = 2.719, *P* = 0.1072; group × drug, *F*_(1,39)_ = 8.609, *P* = 0.0056) as indicated by reduction in CPP scores; no effect of Rox in HIP (Veh-Sal, *n* = 12; Veh-Mor, *n* = 14; Rox-Sal, *n* = 11; Rox-Mor, *n* = 14; group, *F*_(1,47)_ = 39.28, *P* < 0.0001; drug, *F*_(1,47)_ = 0.5163, *P* = 0.4760; group × drug, *F*_(1,47)_ = 0.0995, *P* = 0.7538). **f** The paradigm of Mor-CPP in AAV-infection mice. AAV9-hSyn-EGFP- NC and neuronal specific AAV9-hSyn-EGFP-shHIF-1α were injected into PFC 21 days before CPP performance, Rox (20 μmol/L, 2 μL per site) was injected 2 h before morphine injection every day in conditioning phase. **g** Knockdown of HIF-1α abolished Rox efficiency in inhibiting Mor-CPP acquisition (NC-Veh-Mor, *n* = 10; sh-HIF-1α-Veh-Mor, *n* = 10; NC-Rox+Mor, *n* = 9; sh-HIF-1α-Rox+Mor, *n* = 11; group, *F*_(1,36)_ = 3.829, *P* = 0.0581; drug, *F*_(1,36)_ = 7.412, *P* = 0.0099; group × drug, *F*_(1,36)_ = 4.404, *P* = 0.0429). Values are presented as means ± SEM. Statistical analysis for a, and for b-e and g were performed using one-way ANOVA and two-way ANOVA followed by Bonferroni-corrected tests, respectively. *P < 0.05, **P < 0.01, ***P < 0.001; NS: not significant

In order to clarify the specific brain region(s) involved in Rox-induced inhibition of CPP acquisition, we infused Rox locally into PFC (ACC area), STR, NAc (shell) or hippocampus (HIP). The efficacy of Rox-elevated HIF-1α in representative brain regions (PFC, NAc, STR and HIP) was verified by immunoblot (Fig. 3a and Supplemental Fig. 6). 20 μmol/L Rox was infused in respective brain region with 2 h pretreatment. As shown in Fig. 3b-e, Mor-CPP acquisition was markedly inhibited after local administration of Rox into PFC, NAc or STR but not for HIP. It is also noted that local infusion of Rox did not alter basal locomotor activity (Supplemental Fig. 7a-h). In support, we found that local treatment also suppressed Mor-induced p-CREB expression in PFC and NAc, but not in HIP (Supplemental Fig. 8). We also tested the effect of knockdown of HIF-1α on Rox-inhibited Mor-CPP. Local injections of neuronal specific AAV-shHIF-1α into PFC to knockdown HIF-1α expression, the inhibitory effect of Rox treatment on Mor-CPP was absent (Fig. 3f, g). The accurate infusion sites and HIF-1α knockdown efficiency were indicated in Supplemental Fig. 9a, b. Taken together, these data clearly indicate that the effect of Rox is indeed dependent on HIF-1α and local upregulation of HIF-1α in PFC, STR or NAc is sufficient to attenuate Mor-CPP development.

### Upregulation of HIF-1α by Rox stimulates dopamine transporter (DAT) uptake activity via modulating iron homoeostasis

**Fig. 4:**
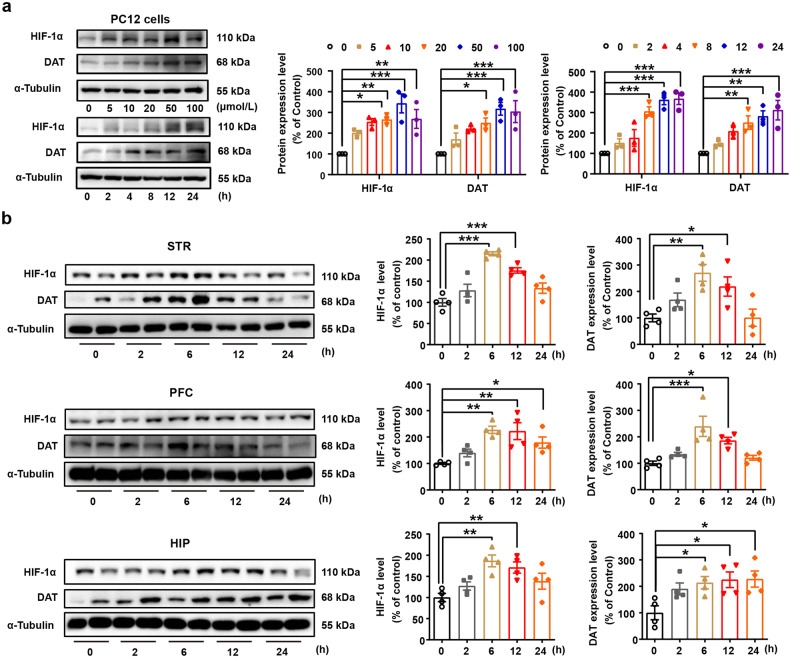
Rox treatment increases dopamine transporter (DAT) expression*.* **a** PC12 cells were treated with roxadustat 24 h at indicated concentration (0–100 μmol/L) or treated with 20 μmol/L roxadustat at indicated time point (0-24 h) for immunoblot assays of HIF-1α and DAT, Rox treatment increased HIF-1α and DAT expression in a dose- and time-dependent manner (Concentration gradient: *n* = 3, drug, *F*_(5,24)_ = 15.65, *P* < 0.0001; protein, *F*_(1,24)_ = 0.4849, *P* = 0.4929; drug × protein, *F*_(5,24)_ = 0.3974, *P* = 0.8457. Time course: *n* = 3, drug, *F*_(5,24)_ = 26.66, *P* < 0.0001; protein, *F*_(1,24)_ = 3.079, *P* = 0.0921; drug × protein, *F*_(5,24)_ = 1.301, *P* = 0.2965). **b** Rox treatment increased HIF-1α and DAT expression in WT mice. WT mice brain tissues (STR, PFC, HIP) were collected after 10 mg/kg roxadustat administration at indicated time point and prepared for immunoblot assays (STR: *n* = 4, HIF-1α, *F*_(4,15)_ = 19.95, *P* < 0.0001; DAT, *F*_(4,15)_ = 6.427, *P* = 0.003. PFC: *n* = 4, HIF-1α, *F*_(4,15)_ = 7.852, *P* = 0.0013; DAT, *F*_(4,15)_ = 8.747, *P* = 0.0008. HIP: *n* = 4, HIF-1α, *F*_(4,15)_ = 6.544, *P* = 0.0029; DAT, *F*_(4,15)_ = 3.987, *P* = 0.0213). Representative images shown in the left panels and quantitative data are shown in the right panels. Values are presented as means ± SEM. Statistical analyses for **a**, and for **b** were performed using two-way ANOVA and one-way ANOVA followed by Bonferroni-corrected tests, respectively. *P < 0.05, **P < 0.01, ***P < 0.001

**Fig. 5:**
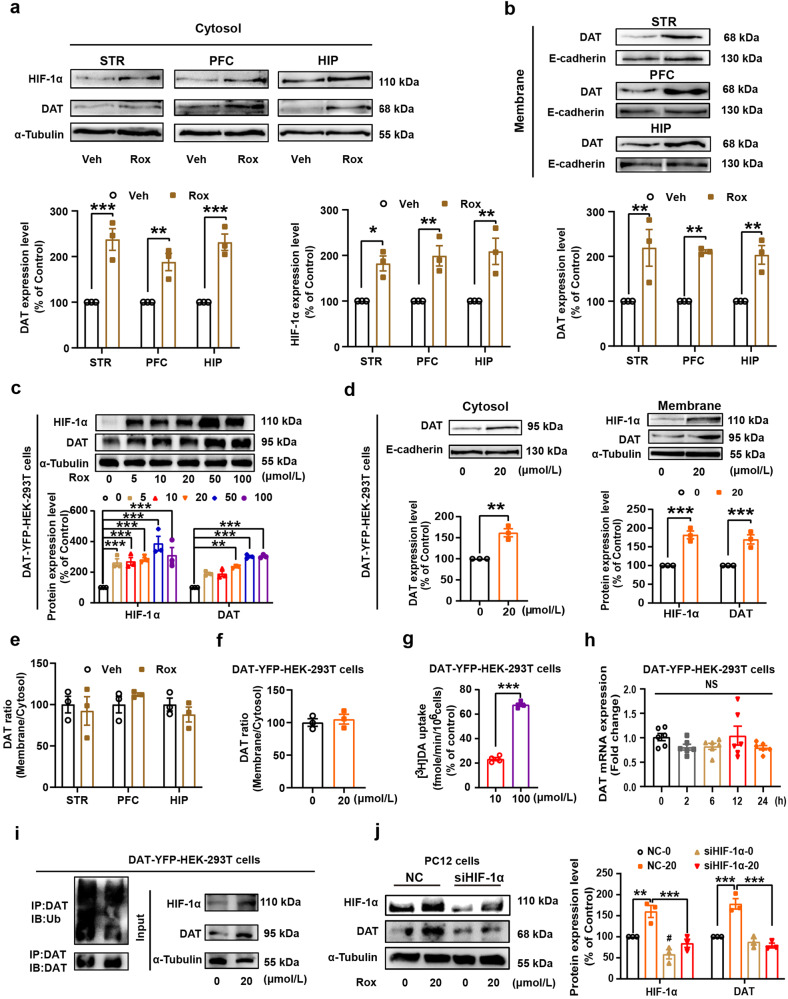
Rox treatment increases plasma DAT expression and uptake activity*.* **a**, **b** WT mice were sacrificed after 6 h roxadustat (10 mg/kg) administration. Then, mice brain tissues (STR, PFC and HIP) were collected and the plasma membrane and cytosol protein were extracted as described in Methods for immunoblot assays. Rox treatment increased DAT expression in plasma and cytosol (**a**: DAT, *n* = 3, drug, *F*_(1,12)_ = 101.7, *P* < 0.0001; area, *F*_(2,12)_ = 1.732, *P* = 0.2184; drug × area, *F*_(2,12)_ = 1.732, *P* = 0.2184; HIF-1α, *n* = 3, drug, *F*_(1,12)_ = 53.13, *P* < 0.0001; area, *F*_(2,12)_ = 0.3386, *P* = 0.7149; drug × area, *F*_(2,12)_ = 0.3386, *P* = 0.7149. **b**: *n* = 3, drug, *F*_(1,12)_ = 52.32, *P* < 0.0001; area, *F*_(2,12)_ = 0.08727, *P* = 0.9170; drug × area, *F*_(2,12)_ = 0.08727, *P* = 0.9170). **c** YFP-tagged DAT stablely expressed HEK-293T (DAT-YFP-HEK-293T) cells were treated with Rox at indicated concentration (0–100 μmol/L) for 24 h and prepared for immunoblot assays. Rox treatment increased HIF-1α and DAT expression in DAT-YFP-HEK-293T cells (*n* = 3, drug, *F*_(5,24)_ = 25.23, *P* < 0.0001; protein, *F*_(1,24)_ = 12.81, *P* = 0.0015; drug × protein, *F*_(5,24)_ = 1.249, *P* = 0.3177). **d** DAT-YFP-HEK-293T cells were treated with 20 μmol/L roxadustat for 24 h. Then, the plasma membrane and cytosol protein were extracted as described in Methods and prepared for immunoblot assays. Rox treatment increased expression of DAT in both plasma and cytosol and HIF-1α in cytosol in DAT-YFP-HEK-293T cells (membrane, *n* = 3, *t* = 6.521, *df* = 4, *P* = 0.0029. cytosol, *n* = 3, drug, *F*_(1,8)_ = 90.17, *P* < 0.0001; protein, *F*_(1,8)_ = 0.5780, *P* = 0.4689; drug × protein, *F*_(1,8)_ = 0.5780, *P* = 0.4689). **e**-**f** Rox treatment did not change the ratio of plasma membrane and cytosol DAT in mice brain tissues as well as in DAT-YFP-HEK-293T cells (**e** *n* = 3, drug, *F*_(1,12)_ = 0.092, *P* = 0.7667; area, *F*_(2,12)_ = 0.7726, *P* = 0.4835; drug × area, *F*_(1,12)_ = 0.7726, *P* = 0.4835. **f** *n* = 3, *t* = 0.5311, *df* = 4, *P* = 0.6235). **g** [^3^H] DA uptake assays in DAT-YFP-HEK-293T cells treated with 10 μmol/L or 100 μmol/L of Rox for 24 h. Rox treatment increased DAT uptake activity as indicated by increased [^3^H] DA uptake (*n* = 4, *t* = 24.21, *df* = 6, *P* < 0.0001). **h** 20 μmol/L Rox trea*t*ment for 0–24 h did not alter DAT mRNA expression in DAT-YFP-HEK-293T cells (*n* = 6, *F*_(4,25)_ = 1.391, *P* = 0.2658). **i** Rox treatment (20 μmol/L, 24 h for cells and 10 mg/kg, 6 h for mouse) inhibited DAT ubiquitination. DAT-YFP-HEK-293T cell lysates were precipitated with DAT antibody-conjugating agarose beads to purify DAT proteins. Followed by immunoblotting with anti-ubiquitin and anti-DAT antibody to detect the ubiquitination levels of DAT and DAT expression in the purified samples, respectively. Total cell lysate was used to detect the total expression of HIF-1α, DAT and α-Tubulin by immunoblotting with specific anti-HIF-1α, anti-DAT and anti-α-Tubulin antibodies. **j** HIF-1α siRNA (50 pmol/L) was transfected into PC12 cells for 48 h to knockdown HIF-1α as described in Methods. Then, PC12 cells were treated with roxadustat (20 μmol/L) for another 24 h before collection for immunoblot assays. Knockdown of HIF-1α by siRNA transfection abolished Rox efficacy in upregulation of HIF-1α and DAT (*n* = 3, group, *F*_(3,16)_ = 45.42, *P* < 0.0001; protein, *F*_(1,16)_ = 2.698, *P* = 0.1200; group × protein, *F*_(3,24)_ = 1.566, *P* = 0.2366). Representative images for **a**-**d** are shown in the upper panels and quantitative data are shown in the lower panels. Representative images for **j** are shown in the left panels and quantitative data are shown in the right panels. Values are presented as means ± SEM. Statistical analyses for **a-c**, **d** (cytosol), **e** and **j**, and for **d** (membrane) and **f**-**g**, and for **h** were performed using two-way ANOVA followed by Bonferroni-corrected tests and Student’s *t*-test and one-way ANOVA followed by Bonferroni-corrected tests, respectively. *P < 0.05, **P < 0.01, ***P < 0.00; NS: not significant

Morphine is known to elicit disinhibition of GABAergic input onto dopaminergic neurons and leads to enhancement of DA release that contributes to the development of morphine use disorder.^37^ DAT acts as an important presynaptic regulator of dopaminergic activity. To further investigate the mechanisms underlying Rox-regulated Mor reward, we studied the effect of Rox on DAT activity. We found that Rox treatment increased DAT expression, both in PC12 cells and in mouse brain regions, and these actions occurred in coordination with elevated expression of HIF-1α (Fig. 4a, b). Increased DAT expression in response to Rox treatment was observed in both plasma membrane fractions and cytosol preparations from mouse brain regions without change in ratio of plasma membrane and cytosol DAT and also in DAT-YFP-HEK-293T cells (Fig. 5a-f). To verify the functional role of Rox-upregulated DAT in plasma membranes, dopamine uptake capacity was determined using a [^3^H] DA radioisotope uptake assay. As expected, Rox increased DAT dopamine uptake capacity (increased by 23.3 ± 0.80% at 10 μmol/L Rox and 67.6 ± 0.80% at 100 μmol/L Rox, relative to controls) in plasma membrane preparations of DAT-YFP-HEK-293T cells (Fig. 5g). It is notable that this increase in DAT expression was not accompanied by increase in mRNA expression in DAT-YFP-HEK-293T cells, indicating that post-translational mechanisms may be involved (Fig. 5h). DAT is known to be subject to dynamic recycling in which ubiquitin modification of DAT is critical for either membrane DAT internalization or its ubiquitin degradation.^4,32^ We found that Rox induced a marked decrease in DAT-ubiquitin conjugates in DAT-YFP-HEK-293T cells (Fig. 5i), indicating that impaired ubiquitin processing of DAT may be responsible for upregulation of DAT expression in response to ROX. Moreover, Rox-stimulated DAT upregulation was abolished by knockdown of HIF-1α using siRNA in PC12 cells, indicating that elevated DAT expression by ROX was dependent on the HIF-1α upregulation (Fig. 5j).

**Fig. 6:**
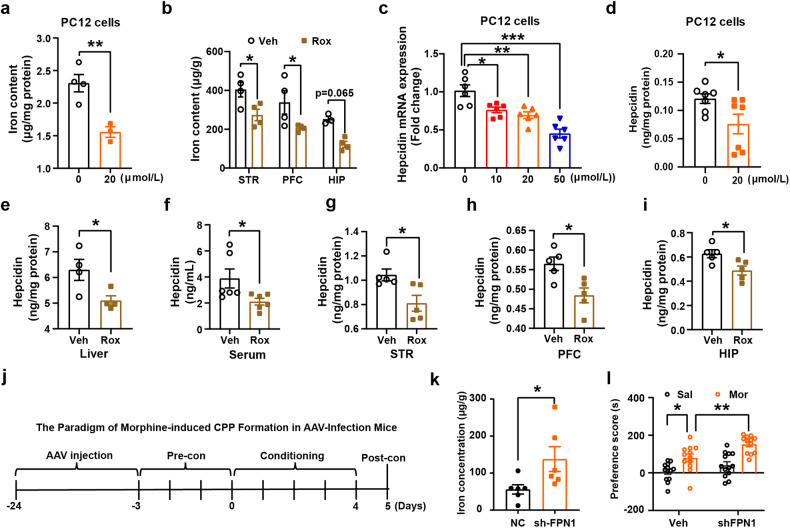
Rox treatment inhibits iron accumulation by reducing hepcidin expression and knockdown of FPN1 induces susceptibility of Mor-CPP. **a** PC12 cells were treated with 20 μmol/L roxadustat for 24 h. Then, the cells were prepared for ICP-MS detection of total iron content. Rox treatment lowered iron content in PC12 cells (0 μmol/L, *n* = 4, 20 μmol/L, *n* = 3, *t* = 4.382, *df* = 5, *P* = 0.0071). **b** WT mice were sacrificed after 6 h roxadustat (10 mg/kg) injection, the brain tissues, liver and serum were collected for ICP-MS detection of total iron content. Rox injection lowered iron content in STR (*n* = 4), PFC (*n* = 4) and HIP (Veh, *n* = 3, Rox, *n* = 4, drug, *F*_(1,17)_ = 21.65, *P* = 0.0002; area, *F*_(2,17)_ = 9.455, *P* = 0.0017; drug × area, *F*_(2,17)_ = 0.0007598, *P* = 0.9992). **c**, **d** PC12 cells were treated with 20 μmol/L Rox for 24 h. Then, the cells were prepared for q-PCR and Elisa assays, Rox treatment lowered the expression of hepcidin in PC12 cells (**c,** *n* = 6, *F*_(3,20)_ = 17.53, *P* < 0.0001. d, *n* = 7, *t* = 2.310, *df* = 12, *P* = 0.0395). **e**-**i** ELISA of hepcidin protein in Rox (10 mg/kg, 6 h) treated WT mice. Rox treatment lowered the expression of hepcidin in mouse liver (*n* = 4, *t* = 2.648, *df* = 6, *P* = 0.0381), serum (*n* = 6, *t* = 2.312, *df* = 10, *P* = 0.0433), STR (*n* = 5, *t* = 2.868, *df* = 8, *P* = 0.0209), PFC (*n* = 5, *t* = 3.169, *df* = 8, *P* = 0.0132), and HIP (*n* = 5, *t* = 2.807, *df* = 8, *P* = 0.0229). **j** The paradigm of Mor-CPP in AAV-infection mice. pAKD-CMV-bGlobin-mCherry-3*FLAG-WPRE-H1-shRNA and pAAV-CBG-mCherry-3*FLAG-WPRE-H1-shFPN1 virus were injected into NAc for 21 days before CPP performance. **k**, **l** After 21 days virus infection, mice brain tissues were collected and prepared for iron content detection with ICP-MS, FPN1 knockdown induced iron accumulation in NAc (*n* = 6, *t* = 2.294, *df* = 10, *P* = 0.0447). FPN1 knockdown mice were more sensitive to Mor-CPP as evidenced by higher CPP scores in shFPN1+Mor group as compared with Veh+Mor group. (NC-Sal, *n* = 13; NC-Mor, *n* = 12; sh-FPN1+Sal, *n* = 13; sh-FPN1+Mor, *n* = 12; group; *F*_(1,46)_ = 10.96, *P* = 0.0018, drug, *F*_(1,46)_ = 32.08, *P* < 0.0001; group × drug, *F*_(1,46)_ = 1.313, *P* = 0.2578). Values are presented as means ± SEM. Statistical analyses for **a** and **d**–**i** and **k**, and for **b** and **l**, and for **c** were performed using Student’s *t*-test and two-way ANOVA and one-way ANOVA followed by Bonferroni-corrected tests, respectively. *P < 0.05, **P < 0.01, ***P < 0.001

Since HIF-1α is known to be involved in regulation of iron metabolism, we recently also reported that alteration of iron homeostasis is associated with DAT ubiquitin modification.^32^ We next investigated the role of iron in Rox-induced attenuation of Mor-CPP development. We found that Rox treatment decreased iron content both in PC12 cells and in mouse brain regions (Fig. 6a, b). To further elucidate the mechanism for Rox-induced iron content decrease, we next analyzed the expression of key regulators of iron homeostasis. Upregulation of HIF-1α by Rox treatment did not alter mRNA expression of iron import-related transporters, such as DMT1 and TfR1 in PC12 cells (Supplemental Fig. 10a, b). However, we detected a decrease in hepcidin, an iron-negative regulatory factor that binds to FPN1 to inhibit exporting activity for iron (Fig. 6c, d). Decreased expression of hepcidin was also observed in mouse liver and peripheral serum, as well as brain regions that included STR, PFC and HIP (Fig. 6e-i).

In order to clarify the role of FPN1, we use AAV virus to locally knockdown FPN1 expression in NAc, the mice were than subjected to Mor-CPP training as indicated in Fig. 6j. The mice with FPN1 knockdown were more sensitive to develop into Mor-CPP as evidenced by CPP scores between the shFPN1-Mor group and Veh-Mor group which may attribute to the increases in iron content (Fig. 6k, l and Supplemental Fig. 9c). These results demonstrated that Rox decreased cellular iron content via upregulation of HIF-1α-stimulated iron exporter (FPN1) activity.

### Mice with iron deficiency fail to develop methamphetamine- and morphine-induced CPP

**Fig. 7:**
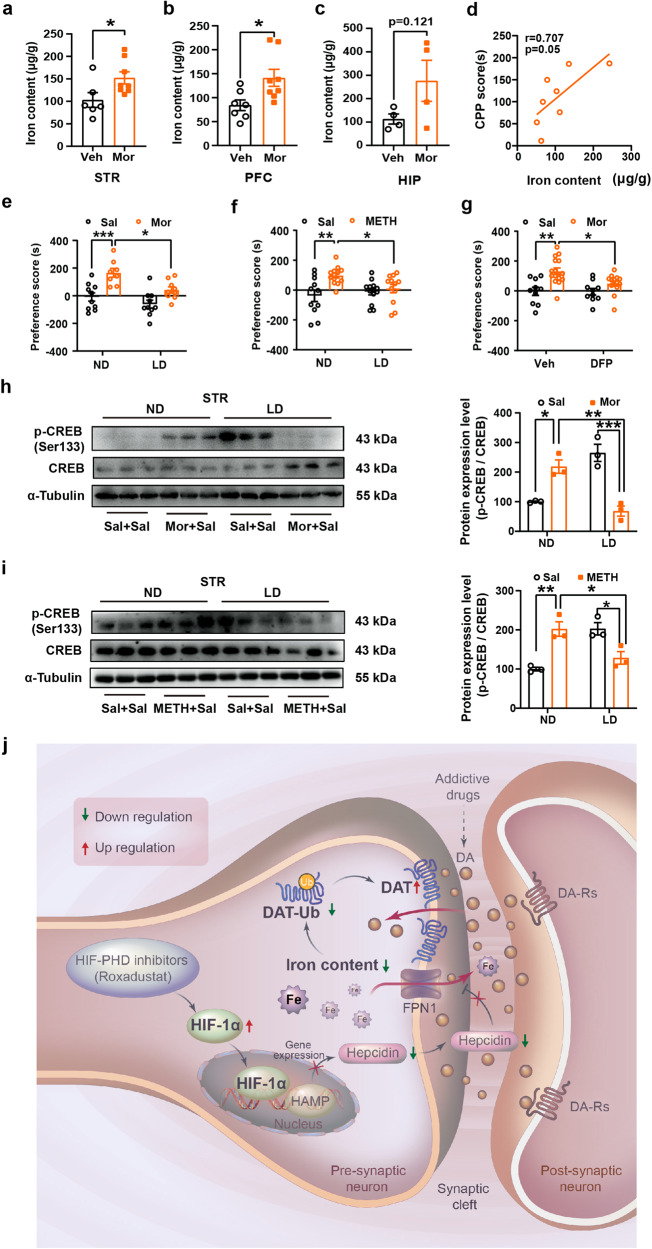
Iron deficiency inhibits Mor- and METH-induced CPP formation and phosphorylation of CREB. **a**-**c** Mouse brain tissues were collected within 24 h after Mor-CPP tests and prepares for ICP-MS detection of total iron content. Iron content was increased after Mor-CPP formation (**a**, Veh, *n* = 6, Mor, *n* = 8, *t* = 2.346, *df* = 12, *P* = 0.0370. **b** Sal, *n* = 7, Mor, *n* = 8, *t* = 2.628, *df* = 13, *P* = 0.0209. **c** Sal, *n* = 4, Mor, *n* = 4, *t* = 1.806, *df* = 6, *P* = 0.1209). **d** The correlation of iron content and CPP score was analyzed by correlation analysis. Iron content was correlated positively with Mor-CPP scores (*n* = 8, *r* = 0.707, *P* = 0.05). **e**, **f** WT mice were fed with low iron diet for 4 weeks as described in Methods. Low iron diet suppressed Mor- and METH-CPP acquisition as indicated by reduction in CPP scores (**e**, ND-Sal, *n* = 10, ND-Mor, *n* = 8, LD-Sal, *n* = 10, LD-Mor, *n* = 9; group, *F*_(1,33)_ = 23.61, *P* < 0.0001; diet, *F*_(1,33)_ = 9.514, *P* = 0.0041; group × diet, *F*_(1,33)_ = 1.616, *P* = 0.2125. **f** ND-Sal, *n* = 11, ND-METH, *n* = 13, LD-Sal, *n* = 13, LD-METH, *n* = 12; group, *F*_(1,45)_ = 9.155, *P* = 0.0041; diet, *F*_(1,45)_ = 1.302, *P* = 0.2598; group × diet, *F*_(1,45)_ = 4.769, *P* = 0.0342). **g** The bilateral cannula was implanted into PFC of mice brain 7 days prior to CPP performance, deferiprone (DFP) (13 mg/mL, 2 μL per site) was injected 6 h before morphine injection every day in conditioning phase. DFP pretreatment suppressed Mor-CPP acquisition as indicated by reduction in CPP scores (Veh-Sal, *n* = 10, Veh-Mor, *n* = 16, DFP-Sal, *n* = 10, DFP-Mor, *n* = 14; group, *F*_(1,46)_ = 18.14, *P* = 0.0001; drug, *F*_(1,46)_ = 3.751, *P* = 0.0589; group × diet, *F*_(1,46)_ = 3.167, *P* = 0.0817). **h**, **i** Mice whole striatum tissues were collected 24 h after Mor- and METH-CPP tests for immunoblot assays, respectively. Low iron diet suppressed Mor- and METH-induced expression of (Ser133) phosphorylated-CREB in STR (**h**, n = 3, group, *F*_(1,8)_ = 3.749, *P* = 0.0889; drug, *F*_(1,8)_ = 0.1284, *P* = 0.7293; group × drug, *F*_(1,8)_ = 61.05, *P* < 0.0001. **i** *n* = 3, group, *F*_(1,8)_ = 0.8998, *P* = 0.3706; drug, *F*_(1,8)_ = 0.9758, *P* = 0.3522; group × drug, *F*_(1,8)_ = 35.83, *P* = 0.0003). **j** Schematic diagram of possible mechanisms underlying Rox-induced amelioration of drug dependence. Rox induces HIF-1α accumulation and nuclear translocation, thereby inhibiting hepcidin transcription. Iron efflux is enhanced by reduction of hepcidin mediated-FPN1 degradation. Finally, iron deficiency suppresses DAT ubiquitin degradation, thus decreasing DA concentration in the synaptic cleft and ultimately ameliorating drug dependence. The Schematic diagram was drawn by Adobe Illustrator software (Version 2022). Representative images for immunoblots are shown in the left panels and quantitative data are shown in the right panels. Values are presented as means ± SEM. Statistical analyses for **a**-**c**, and for **d**, and for **e**-**i** were performed using Student’s *t*-test and correlation analysis and two-way ANOVA followed by Bonferroni-corrected tests, respectively. *P < 0.05, **P < 0.01, ***P < 0.001

So far, we have demonstrated that Rox treatment inhibits morphine reward via disrupting iron homeostasis and upregulating DAT expression. To further elicit the role of iron in drug reward, we first explored the relationship between Mor reward and brain iron content and found brain iron content was elevated in mice with Mor-CPP (Fig. 7a-c). Moreover, we found that brain iron content was positively correlated with Mor-CPP score (Fig. 7d). We next investigated how change in iron content would affect reward behaviors. Using a low iron diet-induced systemic iron deficiency mouse model,^38^ we confirmed that the 4-week low-iron diet induced iron deficiency in mice, in which brain tissues such as STR, PFC and HIP, liver, and peripheral blood all exhibited decreased iron content, without alteration in body weight or induction of anemia (Supplemental Table S1 and Supplemental Fig. 11a-d). Mor- or METH-induced CPP was then conducted in the iron-deficient mice. The results demonstrated that iron deficiency attenuated Mor- and METH-CPP acquisition (Fig. 7e, f); which mimicked the effect of Rox on METH- and Mor-induced CPP. In agreement with previous study that DAT expression was upregulate in mice with low iron diet fed,^39^ we confirmed that plasma DAT expression in brain was also increased in that iron deficient mice (Supplemental Fig. 11e). To further confirm the role of iron deficiency on morphine reward, we applied deferiprone (DFP), a well-known iron chelator to low local iron content in PFC (mainly ACC area) before conducting Mor-CPP (Supplemental Fig. 12a, b). The results showed that local application of DFP into PFC readily inhibited Mor-CPP acquisition (Fig. 7g); this result mimicked the effect of local injection of Rox into PFC. In support, both Mor and METH treatments failed to upregulate p-CREB in iron-deficient mice (Fig. 7h, i). Furthermore, it is notable that diet-induced iron deficiency did not alter basal learning and memory skills in mice (Supplemental Fig. 13a-i). Also, DFP local injection did not alter basal locomotor activity (Supplemental Fig. 12c). These results provide direct experimental evidence that decreased iron concentration suppresses the development of Mor- and METH-mediated reward effects.

## Discussion

In summary, the main findings of the present studies are as follows: (1) Upregulation of HIF-1α by systemic administration of Rox produced robust amelioration of drug reward as evidenced by inhibition of Mor-CPP acquisition, promotion of CPP extinction and inhibition of CPP expression and reinstatement, which indicated that Rox has potential preventive and therapeutic effects for substance use disorder. (2) Mechanistically, HIF-1α activation by ROX treatment resulted in intracellular iron deficiency in brain via downregulation of hepcidin expression, which lead to the enhanced iron exporter via FPN1. Moreover, iron deficiency in response to Rox treatment impaired the iron-dependent ubiquitinated degradation of DAT and resulted in the enhancement of DAT function, which ultimately leads to inhibition of the reward. The present work thus provides direct experimental evidence to indicate the importance of iron homeostasis in drug reward and reveals that Rox may be a novel therapeutic approach for substance use disorder.

HIF-1α is an oxygen-sensitive subunit of hypoxia-induced factor that is degraded by prolyl hydroxylase domain (PHD), which hydroxylates proline residues on HIF-1α in the presence of oxygen. As an oxidative stress sensor, HIF-1α is involved in many biological processes in response to systemic oxygen levels.^40–45^ Rox is a first PHD inhibitor to stimulate HIF-1α expression and activity and was approved by FDA in 2018 for the treatment of anaemia in patients with chronic kidney disease and in patients with myelodysplastic syndromes. This drug was shown to cross the blood-brain-barrier^46,47^ and this property renders it valuable tool for exploring the potential effects of Rox in neurological and psychiatric disorders.^48–50^ Iron overload is considered to contribute to pathogenesis of Parkinson’s disease. It is interesting to noted that administration of similar dose of Rox was shown to protect dopaminergic neurons in mouse models of Parkinson’s disease.^50^ Whether the finding of ROX treatment- induced intracellular low iron contributes to the observed protection on dopaminergic neurons is worth to be confirmed. This drug in 10–20 mg/kg was also shown to exert robust antidepressant activity in chronic unpredictable mild stress model by promoting hippocampal neurogenesis and synaptic plasticity.^49^ Iron is the most abundant transition metal in the brain and important for various neuronal activity, and is known to involve in the regulation of synaptic plasticity.^51^ Whether or how Rox-altered iron homeostasis has anything to do with the drug’s antidepressant effect remained unknown. Alteration in iron homeostasis is associated with ferroptosis. It was reported that spinal cord ferroptosis contributes to morphine tolerance.^51^ A recent study has reported that Rox could inhibit ferroptosis and kidney injury.^23^ Whether Rox-modulated ferroptosis contribute to CPP and drug reward is worth of further investigating.

Clinical studies have revealed that there are change in iron metabolism in substance use disorders including alcohol abuser.^28–31,52,53^ A study conducted in chronic cocaine user revealed that iron content was significantly higher in basal ganglia reward regions than that of control individuals.^54^ It was also reported that iron mediates morphine -induced ferritin heavy chain upregulation.^55^Mice with mitochondrial coq7 mutant had decreased iron content in striatum and hippocampus that is associated with attenuation development of METH- induced CPP. ^56^ In the present study, we found that a Rox produced a promising inhibition on Mor- or METH-reward behavioral. We provide clear experimental evidences to demonstrate that activation of HIF-1α by ROX decreased iron content in brain that impaired DAT-ubiquitin. The elevated DAT functional expression may be underlined the ROX-attenuated Mor- or METH- CPP. Our data may implicate a promising approach for the treatment of substance use disorder by targeting HIF-1α/iron. However, it should be noticed that, although the dose (10 mg/kg) we used here is in the safe range of clinical application for renal anemia, the true translational value the present finding on ROX requires to be tested in clinical proof-of-concept study.

The major finding of the present work is that we provided detailed experimental evidence for the importance of iron homeostasis in the modulation of drug reward development. We demonstrated that Rox promotes the nuclear translocation of HIF-1α (Supplemental Fig. 1c, d), subsequently disturbed hepcidin/FPN1 axis-mediated iron homeostasis, and led to low intracellular iron levels (Fig. 6a-i). It is known that cellular iron homeostasis is subject to precise regulation by importing and exporting components.^57–59^ We did not detect changes in importers of iron but did find enhancement in exporter activity. Hepcidin is known to bind to iron exporter FPN1 and inhibits its activity by modulating internalization and degradation of FPN1. Decrease in hepcidin will result in disinhibition of FPN1 and lead to enhancement of iron exporting activity, ultimately decreased intracellular iron content.^59–63^ We found that Rox treatment decreased cellular iron content, accompanied by decreased hepcidin levels both in PC12 cells and in mice (Fig. 6a-i), which we confirmed that upregulation of HIF-1α by Rox mediated the decreased hepcidin.^60,61^ In agreement with our data, a previous report showed that iron-deficient rats induced by low iron diet increased DAT expression.^39^ We further elucidated that upregulation of HIF-1α-induced cellular iron loss resulted in inhibition of DAT internalization that was attributed to impaired ubiquitin degradation, ultimately leading to DAT accumulation and a functional increase in uptake activity on DA in the plasma membrane (Fig. 5a-i). This is in line with our previous observation in Clk1 mutant mice, in which elevated HIF-1α expression in response to Clk1 deficiency also stimulated DAT expression in the plasma membrane.^32^ It is therefore concluded that upregulation of HIF-1α by Rox-stimulated DAT functional expression via HIF-1α-regulated iron homeostasis is likely responsible for this drug’s effect on drug reward. In addition, we charted that ROX regulates HIF-1α-hepcidin-FPN-iron-DAT pathway, we also found that ROX treatment failed to activate CREB in Mor-CPP, it will be interested to test if the impaired activation of CREB is attributed to the decreased dopamine activity by upregulation of DAT expression in response to ROX treatment.

It should be mentioned that we only employed CPP to assess the effect of Rox on Mor- or METH- reward. It is general believed that morphine disinhibits GABAergic projections onto dopaminergic neurons and facilitates dopamine release are underling mechanism for reward development. We did not test the effect of Rox on Mor-dependence. It was reported that dopamine is not required for the maintain of heroin self-administration,^64^ it will be interested to test if Rox also inhibit Mor-dependence.

In summary, the present work demonstrates that upregulation of HIF-1α by the HIF-PDH inhibitor Rox inhibited drug dependence-related behaviors, including inhibition of CPP acquisition, expression, reinstatement and promotion of CPP extinction. We reveal that elevated HIF-1α expression results in alterations in hepcidin/ FPN1 activities and disruption of iron hemostasis; this leads to elevation in DAT functional expression by inhibition of DAT ubiquitin and degradation, and ultimately results in decreased DA content in the synaptic cleft and attenuation in development of drug reward (Fig. 7j). These findings indicate a potentially novel mechanism for how HIF-1α and iron are involved in substance use disorder. These findings may also repurpose Rox in terms of a new indication for the translational study of substance abuse disorder.

## Materials and methods

### Animals

Male C57BL/6 J mice, age 6–8 weeks (22 ± 3 g), were obtained from Shanghai SLAC Laboratory Animal Co. Ltd. (Shanghai, China). Mice were housed at 4–6 animals per cage, with food and water available ad libitum, in humidity- and temperature-controlled rooms on a 12-hour light/dark cycle (lights on/off at 8:00 am/20:00 pm each day). All animal experiments were performed according to the Animal Care and Use of Soochow University and protocols were proved by its Animal Welfare Committee according to Guidelines for the Care and Use of Laboratory Animals (Chinese National Research Council, 2006). All animals were studied at age 6–8 weeks, with every effort made to minimize animal suffering and the number of animals used.

### Cell culture

PC12 cells were obtained from American Type Culture Collection (ATCC, Manassas, VA, USA). YFP-tagged DAT stable expressed HEK-293T (DAT-YFP-HEK-293T) cells were prepared in the laboratory as previously described.^65^ Cells were cultured in Dulbecco’s modified Eagle’s medium (Gibco, Grand Island, NY, USA) containing 10% fetal bovine serum (FBS) and 1% penicillin/streptomycin.

### Drugs and reagents

Roxadustat (FG-4592) was purchased from Selleck (Shanghai, China) and dissolved in a mixed solvent containing 5% DMSO, 40% PEG-300, 5% Tween-80 and 50% ddH_2_O according to Selleck in vivo usage instructions for mouse intraperitoneal injection. Rox powder was stored at −20 °C in silver paper to avoid light and ensure dryness, with its solvent prepared immediately prior to use. Rox was dissolved in solvent to reach a concentration of 100 mM and then diluted to concentrations of 5–100 μmol/L with cell culture medium for cell assays. METH was obtained from Shanghai Standard Biotech Co., Ltd. (Shanghai, China) and diluted with saline for i.p. injection (2.0 mg/kg). Morphine was purchased from Shenyang First Pharmaceutical Factory (Shenyang, China) and dissolved in saline for i.p. injection (5.0 or 20.0 mg/kg). Deferiprone (DFP) was purchased from Sigma-Aldrich (St. Louis, MO, USA). Phenylmethanesulfonyl fluoride (PMSF) was purchased from Cell Signaling Technology (Danvers, MA, USA). Other common reagents were purchased from Sinopharm (Beijing, China).

### Behavioral tests

#### Conditioned place preference

The CPP apparatus (Jiliang Ltd., Shanghai, China) contains two distinct visual and textural cue compartments: a dark plexiglass/polyvinyl chloride box with fine wire mesh floor, and a white plexiglass/polyvinyl chloride box with wide grid floor. A removable gate between the two boxes ensured that mice were free or restricted to cross the apparatus during different experimental periods. Time spent in each box was recorded and analyzed through infrared beams and an automated analysis system for baseline and test periods, as previously described.^66^ Animals were habituated in the test room for 60 min before beginning experiments. CPP test procedures were as previously described with minor modifications^32,66,67^ and involved three main phases: pre-test phase, conditioning phase, and post-test phase. In the pre-test phase (3 days), the gate between the two boxes was open and mice were allowed to cross freely between them: 900 s of free moving was recorded and analyzed for baseline preferences and mice that spent more than 630 s or less than 270 s in one side were excluded because of marked unconditioned preference; the remaining mice were divided into groups randomly according to the experimental design. In the conditioning phase, mice were limited to one side of the apparatus to perform conditioning training and received saline, Mor (20.0 mg/kg) or METH (2.0 mg/kg) in the drug-paired side in the morning and received saline in the opposite side in the afternoon, with at least a 6-hour interval between treatments. Mice were trained in the drug-paired box for 30 min during 7 consecutive days for Mor-CPP and 60 min during 4 consecutive days for METH-CPP. In the final phase, the gate between two boxes was removed to allow mice to cross freely. Mice were placed in the dark or light box randomly and time spent in each box was recorded during 900 s of free movement between them. Time spent in the drug-paired box was quantified by the automated analysis system, with preference score calculated as the difference in time spent in the drug-paired compartment between post- and pre-test.

#### Extinction

*Pattern 1 (training extinction):* Mice were trained to establish a CPP model with the method in *Conditioned place preference* section. After the post-test phase, extinction training was performed as described previously.^66^ The gate between the two boxes was closed, mice were injected with saline and placed in the drug-paired box in the morning and then injected with saline and placed in the opposite box in the afternoon. Rox was administered 6 h before the morning training. As for the CPP extinction phase, each extinction training session lasted 30 min with two days of training for a cycle. The extinction session consisted of 3 cycles (Extin-1, Extin-2 and Extin-3). The day after each training cycle, the gate between the two boxes was removed and mice were placed in the dark or light box randomly and time spent in each box was recorded during 900 s of free movement. The time spent in the drug-paired box was quantified by the automated analysis system as preference score for extinction outcomes.

*Pattern 2 (testing extinction):* Mice were trained to establish a CPP model with the method in *Conditioned place preference* section. After the post-test phase, extinction testing was performed as described previously.^67^ The gate between the two boxes was removed, mice were reintroduced into the CPP apparatus randomly on each day for 900 s on 5 consecutive days (Test1–5) without saline or any drug injection and time spent in each box was recorded during 900 s of free movement. The time spent in the drug-paired box was quantified by the automated analysis system as preference score for extinction outcomes.

#### Reinstatement

Following the *training extinction* period, mice received a single injection of saline or a low dose of Mor (5.0 mg/kg) to reinstate Mor-CPP. Mice were placed in the CPP apparatus 10 min following Mor injection and time spent in each box during 900 s of free movement was quantified by the automated analysis system. Preference score was calculated as the difference in time spent in the drug-paired compartment between the reinstatement test and pre-test.

### Western blotting

Cells or animal tissues were lysed in RIPA buffer (Beyotime, Shanghai, China) with PMSF (1 mM, Cell Signaling Technology, Danvers, MA, USA) for 30 min on ice and shocked every 10 min. Then, the samples were centrifuged at 12,500 g for 15 min at 4 °C and the supernatant was collected. BCA Protein Assay Kit (Beyotime, Shanghai, China) was used to detect protein concentration. 5 × loading buffer was added to the remaining supernatant and incubated at 95 °C for 10 min to ensure complete denaturation of protein. Western blotting was performed with the Cell Signaling Technology standard protocol and blots were probed with primary antibodies: anti-HIF-1α (1:1000, Proteintech, Wuhan, China, 20960-1-AP), anti-p-CREB (1:1000, Cell Signaling Technology, Danvers, MA, USA, 9198 L), anti-CREB (1:1000, Cell Signaling Technology, Danvers, MA, USA, 9197 S), anti-Dopamine transporter (1:1000, Abcam, Cambridge, MA, USA, ab184451), anti-E-cadherin (1:5000, Proteintech, Wuhan, China,20874-1-AP), anti-Ubiquitin (1:1000, Santa Cruz, CA, USA, SC-8017), anti-Lamin B1 (1:1000, Proteintech, Wuhan, China, 12987-1-AP), anti-GAPDH (1:1000, Proteintech, Wuhan, China, 10494-1-AP), anti-α-Tubulin (1:10000, Sigma, St. Louis, MO, USA, T6074). After incubation with respective secondary antibodies (Rabbit anti mouse IgG, 1:20000, Sigma, St. Louis, MO, USA, A9044; Goat anti rabbit IgG, 1:20000, Sigma, St. Louis, MO, USA, A0545), the blots were captured by ClinxChemi-Capture 3300 Mini (Clinx Science Instrument, Shanghai, China). At least 3 independent experiments were conducted for each immunoblot assay and blot images were analyzed by Image J software (version 1.52 a).

### Quantitative real time-PCR

Total RNA was extracted from cells using RNAiso Plus (TaKaRa, Tokyo, Japan) according to the manufacturer’s instructions. RNA (1,000 ng) was reverse transcribed into cDNA using the TaRaKa reverse transcription kit with Oligo (dT) primer and cDNA was amplified using the specific primers in Supplemental Table S2. A StepOnePlus^TM^ Real-Time PCR System (Applied Biosystems, Carlsbad, CA, USA) was used to quantify mRNA expression using SYBR Premix II (TaKaRa, Tokyo, Japan). The parameters for quantitative real time-PCR were 30 s at 95 °C, 5 s at 95 °C and 30 s at 60 °C for 40 cycles. GAPDH was used as reference gene and relative quantification of target genes was determined using the 2^-ΔΔCT^ formula. Results were expressed as fold changes relative to the control group as previously described.^32^

### Immunofluorescence

For mouse brain tissue, on completion of behavioral tests mice were anaesthetized with pentobarbital sodium (60 mg/kg i.p.) and then perfused with 40 mL 0.9% saline and subsequently with 40 mL 4% paraformaldehyde in 0.1 mol/L PBS (PH 7.4). Brains were transferred into a 10 mL centrifuge tube containing 4% PFA for a further 6 h for post-fixing at 4 °C and dehydration in a 30% sucrose solution for one week, with the sucrose solution renewed every two days. Dehydrated brains were cut into 20 μmol/L sections using a Leica freezing microtome (Wetzlar, Germany). For cell immunofluorescence, the staining protocol was as described previously.^32^ In brief, PC12 cells were washed 3 times with 0.01 mol/L PBS and fixed with iced methanol for 20 min. For immunofluorescence assay, the target brain sections or fixed PC12 cells were washed three times in 0.1 mol/L PBS. Then, samples were incubated in 0.01 mol/L PBS containing 3% BSA and 0.3% Triton X-100 for 2 h at room temperature. The samples were then incubated with anti-Δfos B antibody (1:1000, Santa Cruz, CA, USA, SC-398595) or anti-HIF-1α antibody (1:1000, Proteintech, Wuhan, China, 20960-1-AP) overnight at 4 °C and washed three times (10 min each time) in PBST (0.1% Triton X-100 in 0.01 mol/L PBS). After washing, samples were incubated with anti-Alexa Fluor 488 conjugated goat anti-rabbit IgG (1:500, Invitrogen, Carlsbad, CA, USA, A11001) or anti-Alexa Fluor 546 conjugated donkey anti-rabbit IgG (1:500, Invitrogen, Carlsbad, CA, USA, A10040) for two hours at room temperature and subsequently washed three times with PBST. Finally, samples were stained with DAPI (1:10000, Thermo Scientific, Waltham, MA, USA) for a further 30 min at 37 °C and again washed three times with PBS. Then, samples were fixed in glass slides and fluorescence observed and captured with a confocal microscope (Zeiss 271 LSM710 META, Jena, Germany).

### Enzyme-linked immunosorbent assay (ELISA)

PC12 cells were homogenized ultrasonically in PBS and centrifuged at 5,000 g for 10 min 4 °C. Then, the supernatant of PC12 cell lysates was collected for measurement of hepcidin protein expression level. For mouse serum, mice were anaesthetized with pentobarbital sodium (60 mg/kg i.p.) and whole blood was collected from the orbit and centrifuged at 1,000 g for 20 min at 4 °C. Then, the supernatant was collected and moved into a new tube. For tissues, the samples were homogenized ultrasonically in 0.1 mol/L PBS containing PMSF (1 mM, Cell Signaling Technology, Danvers, MA, USA) and the supernatant was collected after centrifugation at 5,000 g for 10 min at 4 °C. Protein concentration was measured using BCA Protein Assay Kits (Beyotime, Shanghai, China). Hepcidin ELISA Kits were purchased from Elabscience Biotechnology Co., Ltd (TX, USA). The ELISA procedure was conducted in accordance with manufacturer’s instructions as previously described.^32^ Briefly, 100 μL of sample or standard was added to each ELISA well and incubated for 90 min at 37 °C. Then, the liquid was removed and 100 μL biotinylated detection antibody was added into each well for 60 min at 37 °C. After aspirating and washing three times (2 min each in wash buffer), HRP conjugate (100 μL) was added into each well and incubated for 30 min at 37 °C, followed by aspiration and washing five times with wash buffer. Substrate regent (90 μL) was added into each well and incubated for 15 min at 37 °C. Finally, stop solution (50 μL) was added and the *OD* values measured immediately at 450 nm using a Microplate Reader (Infinite M200 PRO, Tecan, Switzerland).

### Establishment of iron deficiency mouse model

Low iron diet and normal diet were purchased from Jiangsu Xietong Pharmaceutical Bioengineering Co., Ltd (Jiangsu, China). Male C57BL/6 J mice (age 6–8 weeks) were fed with low or normal diet for 3-4 weeks to establish an iron deficiency mouse model^68–70^ before beginning CPP experiments.

### Detection of iron content

Iron content of PC12 cells, mouse tissue or blood samples was detected as previous described.^32^ Briefly, mouse tissues were weighed wet and nitrified with a 5 mL mixed solution containing nitric acid, hydrochloric acid and perchloric acid (1:3:1, v- v- v). PC12 cells were collected and divided into two fractions: one fraction was homogenized ultrasonically in PBS and the protein concentration was detected by BCA Kits (Beyotime, Shanghai, China); the remaining fraction was nitrified by heating to 260 °C as for mouse tissue nitration. Finally, samples were measured by inductively coupled plasma mass spectrometry (ICP-MS) (Thermo Fisher Scientific, Waltham, MA, USA). Iron standard solution (1 mg iron/mL) was purchased from Guobiao Testing & Certification Co., Ltd (Beijing, China), with the standard curve prepared as described previously.^32^

### Membrane protein extraction

Mem-PER™ Plus Membrane Protein Extraction Kit was purchased from Thermo Fisher Scientific Inc (Thermo Scientific, Waltham, MA, USA) and used according to manufacturer’s instructions. For PC12 cells, 5 × 10^6^ cells were collected and washed using cell wash solution. 0.75 mL of Permeabilization Buffer was added to the cell pellet and incubated for 10 min at 4 °C with constant mixing. Collected cells were then centrifuged for 15 min at 16,000 g. Following the addition of 0.5 mL of solubilization buffer to the pellet and incubation at 4 °C for 30 min, with constant mixing, the samples were again centrifuged at 16,000 g for 15 min at 4 °C and the supernatant containing solubilized membrane and membrane-associated proteins was collected. For mouse tissue membrane protein collection, tissues were washed and cut into small pieces with a pair of scissors; 1 mL of permeabilization buffer was added to the tissue and homogenized with a grinding rod. Following addition of 1 mL permeabilization buffer and incubation for 10 min at 4 °C with constant mixing, the sample was centrifuged at 16,000 g for 15 min at 4 °C; the remaining procedures for preparation of cellular fractions were then as described above. Concentration of protein in each preparation was measured using BCA Protein Kits (Beyotime, Shanghai, China).

### Nuclear and cytoplasmic protein extraction

PC12 cells were collected and washed with PBS. Cytoplasmic protein extraction solvent A (400 μL with 1 mM PMSF) was added and incubated for 10 min on ice, followed by addition of 20 μL cytoplasmic protein extraction solvent B for 1 min on ice. After centrifugation at 16,000 g for 5 min at 4 °C, the cytoplasmic protein supernatant was collected. The pellet was added to 100 μL nuclear protein extraction solvent (with 1 mM PMSF) and incubated for 30 min on ice with vortexing for 15 s every 2 min. Then, following centrifugation at 16,000 g for 10 min at 4 °C the nuclear protein supernatant was collected. Protein content of nuclear and cytoplasmic protein supernatants (5 μL) was detected using BCA Protein Kits (Beyotime, Shanghai, China).

### Surgery, cannula implantation and local drug delivery

Mice were anesthetized using pentobarbital sodium (60 mg/kg i.p.) and fixed in a stereotaxic apparatus (RWD Life Science, Shenzhen, China). Mouse body temperature was maintained using a heating pad. Stereotaxic surgery was performed as described previously.^71–74^ Stainless steel guide cannulae was implanted according to the Paxinos and Watson Brain Atlas. Guide cannulae were 0.5 mm shorter than injection needles and placed 0.5 mm above the areas to be targeted. These areas had the stereotaxic coordinates (AP: anteroposterior, ML: mediolateral, DV: dorsoventral): PFC (anterior cingulate cortex, ML = ± 0.3 mm; AP = + 1.0 mm; DV = − 2.0 mm), NAc (nucleus accumbens shell, ML = ± 0.6 mm; AP = + 1.53 mm; DV = − 4.75 mm), STR (dorsomedial striatum, ML = ± 1.35 mm; AP = + 0.85 mm; DV = − 3.1 mm) and HIP (dorsal hippocampus, ML = ± 1.5 mm; AP = − 2.1 mm; DV = − 1.25 mm). The guide cannulas were kept patent with stylets and affixed to the skull using dental cement with two stainless steel screws serving as anchors. Following surgery, animals were housed for at least 3 days before CPP experiments. For Rox microinjection, Rox was dissolved in DMSO at a final concentration of 0–50 μmol/L and injected (2 μL) into the brain regions noted above at 2 h before CPP training. Deferiprone (DFP) was dissolved in PBS at a final concentration of 13 mg/mL and injected (2 μL) into PFC.^75^

### Dopamine uptake assays

YFP-tagged DAT stable expressed HEK-293T (DAT-YFP-HEK-293T) cells were seeded in a 6-well plate and dopamine uptake assays performed as previously described^76,77^ Briefly, DAT-YFP-HEK-293T cells were washed twice with PBS, then 2 mL complete DMEM medium was added to each well, followed by addition of Rox at a final concentration of 10 µM or 100 µM and incubation for a further 24 h. Cells were washed 3 times with PBS and pre-incubated for 30 min in Krebs buffer (118.0 mM sodium chloride, 4.7 mM potassium chloride, 1.2 mM magnesium sulfate, 1.2 mM potassium dihydrogen phosphate, 2.25 mM calcium chloride, 3.25 mM sodium bicarbonate and 11.1 mM glucose, pH 7.2–7.4) containing 10 mM desipramine (to block the endogenous norepinephrine transporter). For uptake assays, [^3^H] DA (3,4-[Ring-2,5,6,-^3^H]-DA; Perkin-Elmer Life Sciences, Boston, MA, USA) was added at a final concentration of 50 nM per well and incubated at 37 °C for 5 min. DA uptake was terminated by immediate removal from the medium and washing 3 times with cold Krebs buffer on ice. Cells were dissolved in 1 mol/L NaOH for 15 min, with shaking, followed by addition of 1 mol/L HCl. Subsequently, all liquid content from each well was collected and radioactivity was determined by liquid scintillation counting (Beckman Instruments, Irvine, CA, USA) using Ecoscint A Cocktail (National Diagnostics, Atlanta, GA, USA). Cells in each well were measured by cell counter, with total protein content of cells measured by BCA Protein Kits (Beyotime, Shanghai, China).

### siRNA transfection

HIF-1α siRNA was purchased from GenePharma Co., Ltd. (Shanghai, China) with the following sequences: sense 5’-CCAUGUGACCAUGAGGAAATT-3’, antisense 5’-UUUCCUCAUGGUCACAUGGTT-3’; negative control (NC): sense 5’-UUCUCCGAACGUGUCACGUTT-3’, antisense 5’-ACGUGACACGUUCGGAGAATT-3’. siRNA transfection was performed with Lipofectamine 2000 Transfection Reagent (Invitrogen) according to manufacturer’s instructions. PC12 cells were transfected with 30 pM siRNA for 48 h, followed by Rox treatment for another 24 h. Then, knockdown efficiency was validated by immunoblot analysis.

### Stereotaxic injection of adeno-associated virus serotype 9 (AAV9) in mouse prefrontal cortex

AAV9-hSyn-EGFP-shHIF-1α and AAV9-hSyn-EGFP-NC virus particles were provided by Genechem Co., Ltd. (Shanghai, China). pAKD-CMV-bGlobin-mCherry-3*FLAG-WPRE-H1-shRNA and pAAV-CBG-mCherry-3*FLAG-WPRE-H1-shFPN1 virus particles were purchased from OBiO Technology Co., Ltd. (Shanghai, China). Surgery was performed under pentobarbital anaesthesia as described in *Sugery, cannula implantation and local drug delivery* section^72^ using the same PFC coordinates: ML = ± 0.3 mm; AP = + 1.0 mm; DV = − 2.0 mm. The virus (1 × 10^12^ viral particles/mL, 200 nL/side) was injected bilaterally into PFC areas at a flow rate of 0.1 μL/min, with the needle retained in position for 10 min after injection to limit viral leakage. CPP testing was performed 3 weeks after virus injection.

### Statistical analysis

Data were analyzed using Graphpad Prism 8.0 (GraphPad Software, Inc. La Jolla, CA, USA). Values are presented as means ± SEM. Differences between multiple groups were determined using one-way or two-way ANOVA followed by Bonferroni-corrected tests. Differences between two groups were determined by two-tailed unpaired Student’s *t*-test. *P* < 0.05 was considered statistically significant.

### Supplementary Information

Supplementary Materials

## Data Availability

All data were available within the manuscript, supplementary materials, or available from the corresponding author upon reasonable request.
