# Supplementary material for: Hypoxia-inducible factor upregulation by roxadustat attenuates drug reward by altering brain iron homoeostasis
Source: Signal Transduct Target Ther. 2023 Sep 18;8:355. doi: 10.1038/s41392-023-01578-2 (PMC10505610; doi:10.1038/s41392-023-01578-2)
Supplement: Supplementary file 1 — Supplementary Materials [file 41392_2023_1578_MOESM1_ESM.docx]

Supplementary Materials for

Hypoxia-inducible factor upregulation by roxadustat attenuates drug reward by altering brain iron homoeostasis

Pengju Yan^1^†, Ningning Li^1^†, Ming Ma^1^†, Zhaoli Liu^1^, Huicui Yang^1^, Jinnan Li^2^, Chunlei Wan^1^, Shuliu Gao^1^, Shuai Li^1^, Longtai Zheng^1^, John L. Waddington^1,3^, Lin Xu^2^*, Xuechu Zhen^1^*

Correspondence to: [zhenxuechu@suda.edu.cn](mailto:zhenxuechu@suda.edu.cn); [lxu@vip.163.com](mailto:lxu@vip.163.com)

**This PDF file includes:**

Materials and Methods

Figures. S1 to S13

Tables S1 to S2

Materials and Methods

**MTT assay**

PC12 cells or HEK-293T cells were seeded in 96-well plate at 4000 cells per well. After 12 hours, different concentrations of roxadustat (Rox) ranging from 0-100 μmol/L were added into each well (three repeated wells for each concentration) and cells further cultured in the cell incubator for another 24 hours. Then, the medium was removed and 30 μL of MTT solution (0.5 mg/ml) was added into each well and the plate was incubated at 37℃ for 3 hours. Then, DMSO (100 μL) was added into each well and the plate was shaken with coverage by silver paper for 10 min. The *OD* values were detected at 570 nm in a Microplate Reader (Infinite M200 PRO, Tecan, Switzerland) within 10 min.

**Locomotor activity**

Locomotor activity assessment and data collection were conducted as previously described with minor modification ^1,2^. Mice were moved to the test room 60 min before starting the experiment. A 20 × 20 × 35 cm plexiglas/polyvinyl chloride box with an infrared photobeam activity system (Jiliang Ltd., Shanghai, China) was used to track and record horizontal movement. Locomotor activity was recorded immediately after saline (Sal), Rox, morphine (Mor) or methamphetamine (METH) administration. Total distance travelled was recorded and analyzed during a 60 min period via the device's automated analysis system. For determination of center time, the trajectory of movement was analyzed for 30 min via the device's automated analysis system.

**Elevated plus maze (EPM)**

Animals were moved to the test room 60 min before starting the experiment. The EPM apparatus (length: width 65 × 5 cm) consists of open arms, enclosed arms and a central platform, made of blue polypropylene, raised 50 cm above the floor and with the enclosed arms having 10 cm walls to ensure a constrained environment. Mice were placed on the central platform with their head towards the enclosed arm and an infrared photobeam system was used to track and record horizontal movement during a 5 min period ^3^. The time and entries of mice in open or enclosed arms were analyzed by the device's automated analysis system.

**Morris water maze**

The water maze consists of a 120 cm diameter circular white plastic tank (Med Associates, Inc., St. Albans, VT, USA) with temperature control to keep water at 21 ± 1℃. The water surface was covered with white polyethylene plastic particles to conceal the platform (10 cm in diameter) underwater. The experimental procedures and data collection were conducted as previously described with minor modifications ^4,5^. Briefly, the test includes three main phases: adaption (1 day), consecutive spatial learning (5 days) and spatial memory testing (1 day). In the adaption phase, mice were placed into the apparatus and allowed to explore freely for 1 min. During the spatial learning phase, mice were placed into the apparatus facing the wall of the water tank. Each mouse was trained in the apparatus for four different quadrants per day with an inter-trial interval of at least 10 min. Mice were allowed to find the hidden platform in 1 min for each trial during this phase; the average time mice spent escaping onto the hidden platform for the four different quadrants was recorded to score spatial learning. In the final phase, the hidden platform was removed, each mouse was released into the water maze from the diagonal quadrant of the original hidden platform and allowed to swim freely for 1 min. The time mice spent in the target quadrant (i.e. original hidden platform quadrant) and platform frequency were recorded to score spatial memory. All data were recorded and analyzed using the EnthoVision 8.0 program (Noldus, Beijing, China).

**Fear conditioning**

Contextual fear conditioning was conducted as described previously with minor modifications ^6-8^. Briefly, the apparatus consists of a conditioning chamber (32 × 25 × 25 cm) inside a ventilated and sound-attenuated box (75 × 64 × 36 cm) (Med Associates, Inc., St. Albans, VT, USA). Each mouse was placed into the inside box and allowed to explore for 5 min before receiving five electrical foot shocks (0.8 mA, 2 s with a 90 s inter-shock interval). After the last shock, the mouse was allowed to stay in the chamber for an additional 2 min prior to return to the home cage. Contextual fear memory was measured at day 2 (Test 1) and day 10 (Test 2) for 5 min. Freezing, defined as the absence of all movement except breathing, was used as an index of fear memory in mice. All data were recorded by the inbuilt digital infrared video camera.

**Reference**

1 Ren, Z. et al. Dihydromyricetin exerts a rapid antidepressant-like effect in association with enhancement of BDNF expression and inhibition of neuroinflammation. *Psychopharmacology* **235**, 233-244 (2018).

2 Ren, Z. X., Zhao, Y. F., Cao, T. & Zhen, X. C. Dihydromyricetin protects neurons in an mptp-induced model of parkinson's disease by suppressing glycogen synthase kinase-3 beta activity. *Acta Pharmacol. Sin.* **37**, 1315-1324 (2016).

3 Walf, A. A. & Frye, C. A. The use of the elevated plus maze as an assay of anxiety-related behavior in rodents. *Nat. Protoc.* **2**, 322-328 (2007).

4 Wang, N. Y. et al. Ferulic acid ameliorates alzheimer's disease-like pathology and repairs cognitive decline by preventing capillary hypofunction in APP/PS1 mice. *Neurotherapeutics* **18**, 1064-1080 (2021).

5 Li, H. B. et al. Antistress effect of TRPV1 channel on synaptic plasticity and spatial memory. *Biol. Psychiatry* **64**, 286-292 (2008).

6 Dai, J. X. et al. Enhanced contextual fear memory in central serotonin-deficient mice. *Proc. Natl. Acad. Sci. U. S. A.* **105**, 11981-11986 (2008).

7 Bai, H. Y., Cao, J., Liu, N., Xu, L. & Luo, J. H. Sexual behavior modulates contextual fear memory through dopamine D1/D5 receptors. *Hippocampus* **19**, 289-298 (2009).

8 Tan, J. W. et al. Impaired contextual fear extinction and hippocampal synaptic plasticity in adult rats induced by prenatal morphine exposure. *Addict. Biol.* **20**, 652-662 (2015).


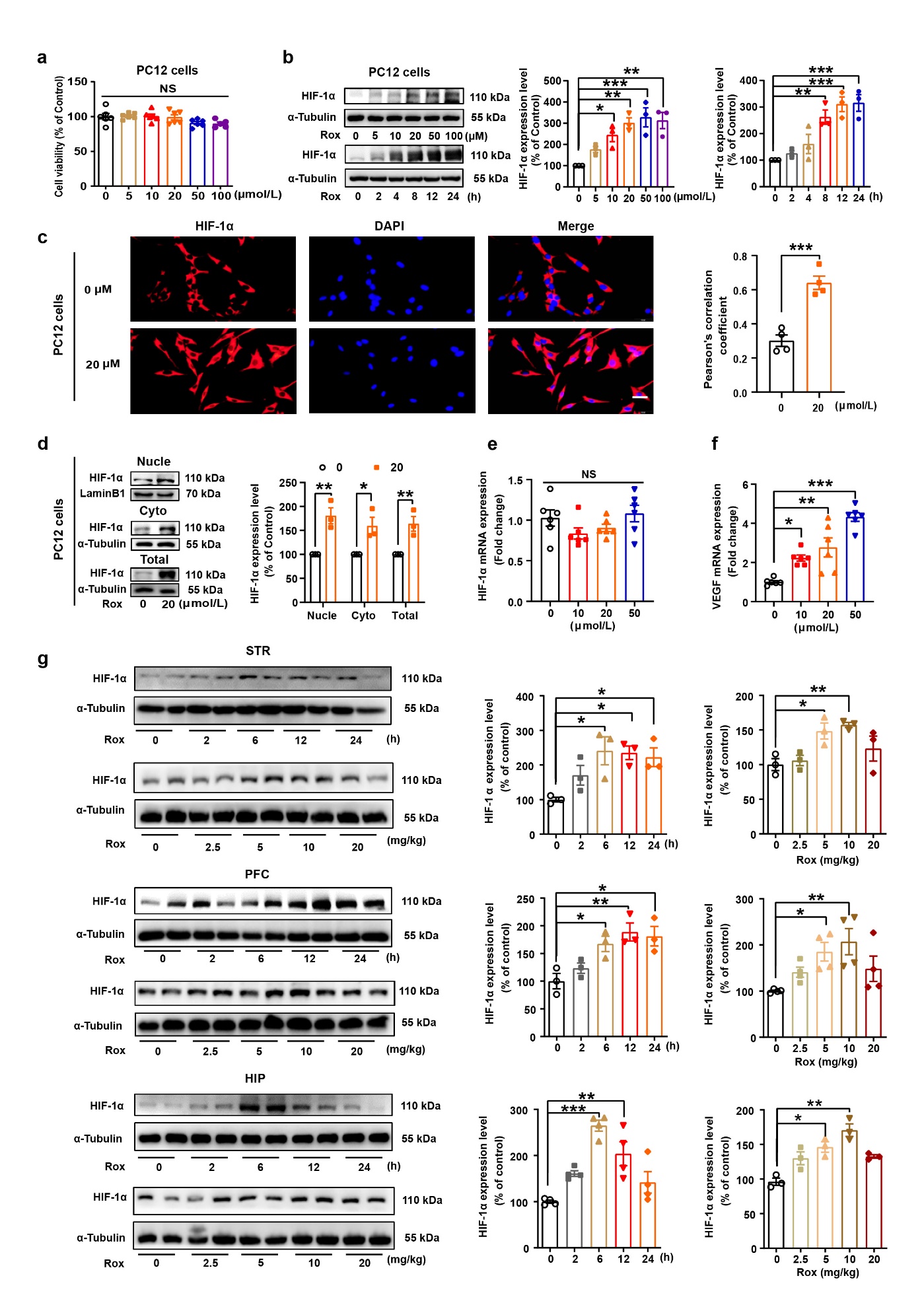
Figure. S1.

**Figure. S1. Roxadustat (Rox) upregulates functional HIF-1α expression *in vitro* and *in vivo*. (a-b)** PC12 cells were treated with Rox for 24 hours at indicated concentration or 20 μmol/L roxadustat for indicated time point for cell viability and immunoblot assays of HIF-1α. Rox treatment increased HIF-1α expression in a dose (0-100 μmol/L for 24 hours)- and time (20 μmol/L for 0-24 hours)-dependent manner without altering cell viability of PC12 cells (**a**, 0 μmol/L, n = 6, 5 μmol/L, n = 6, 10 μmol/L, n = 6, 20 μmol/L, n = 6, 50 μmol/L, n = 6, 100 μmol/L, n = 5, *F*_(5,29)_ = 2.541, *P* = 0.0503; **b**, *n* = 3, *F*_(5,12)_ = 9.202, *P* = 0.0009; time, *n* = 3, *F*_(5,12)_ = 14.11, *P* = 0.0001). **(c-d)** Rox treatment (20 μmol/L, 24 hours) increased HIF-1α nuclear translocation in PC12 cells as indicated by enhanced co-localization in immunofluorescence (scale bar, 100 μmol/L) and nuclear HIF-1α expression in immunoblot assay (**c,** *n* = 4, *t* **=** 6.538, *df* = 6, *P* = 0.0006; **d**, *n* = 3, drug, *F*_(1,12)_ = 48.90, *P* < 0.0001; area, *F*_(2,12)_ = 0.4463, *P* = 0.6502; drug × area, *F*_(2,12)_ = 0.4463, *P* = 0.6502). **(e-f)** mRNA expression of HIF-1α and VEGF was detected by quantitative real-time PCR (q-PCR). Rox treatment for 24 hours increased mRNA expression of vascular endothelial growth factor (VEGF) but not of HIF-1α (**e**, *n* = 6, *F*_(3,20)_ = 1.881, *P* = 0.1654. **f**, *n* = 6, *F*_(3,20)_ = 23.63, *P* < 0.0001). **(g)** WT mice were received a single injection of Rox (10 mg/kg) at indicated time point or received a single injection of Rox at indicated concentration for 6 hours. Then, the brain tissues were collected and processed for immunoblot assays. Rox treatment increased HIF-1α expression in a time- and dose-related manner in mouse striatum (STR), prefrontal cortex (PFC) and hippocampus (HIP) (STR, time, *n* = 3, *F*_(4,10)_ = 4.904, *P* = 0.0189; dose, *n* = 3, *F*_(4,10)_ = 6.555, *P* = 0.0074.; PFC, time, *n* = 3, , *F*_(4,10)_ = 6.244, *P* = 0.0087; dose, *n* = 4, *F*_(4,15)_ = 4.098, *P* = 0.0193. HIP, time, *n* = 4, *F*_(4,15)_ = 14.14, *P* < 0.0001; dose, *n* = 3, *F*_(4,10)_ = 7.514, *P* = 0.0046). Representative images for immunoblot are shown in the left panels and quantitative data are shown in the right panels. Values are presented as means ± SEM. Statistical analyses for **a, b** and **e-g**, and for **c**, and for **d** were performed using one-way ANOVA followed by Bonferroni-corrected tests, Student’s *t*-test and two-way ANOVA followed by Bonferroni-corrected tests, respectively. **P* < 0.05, ***P* < 0.01, ****P* < 0.001; NS: not significant.


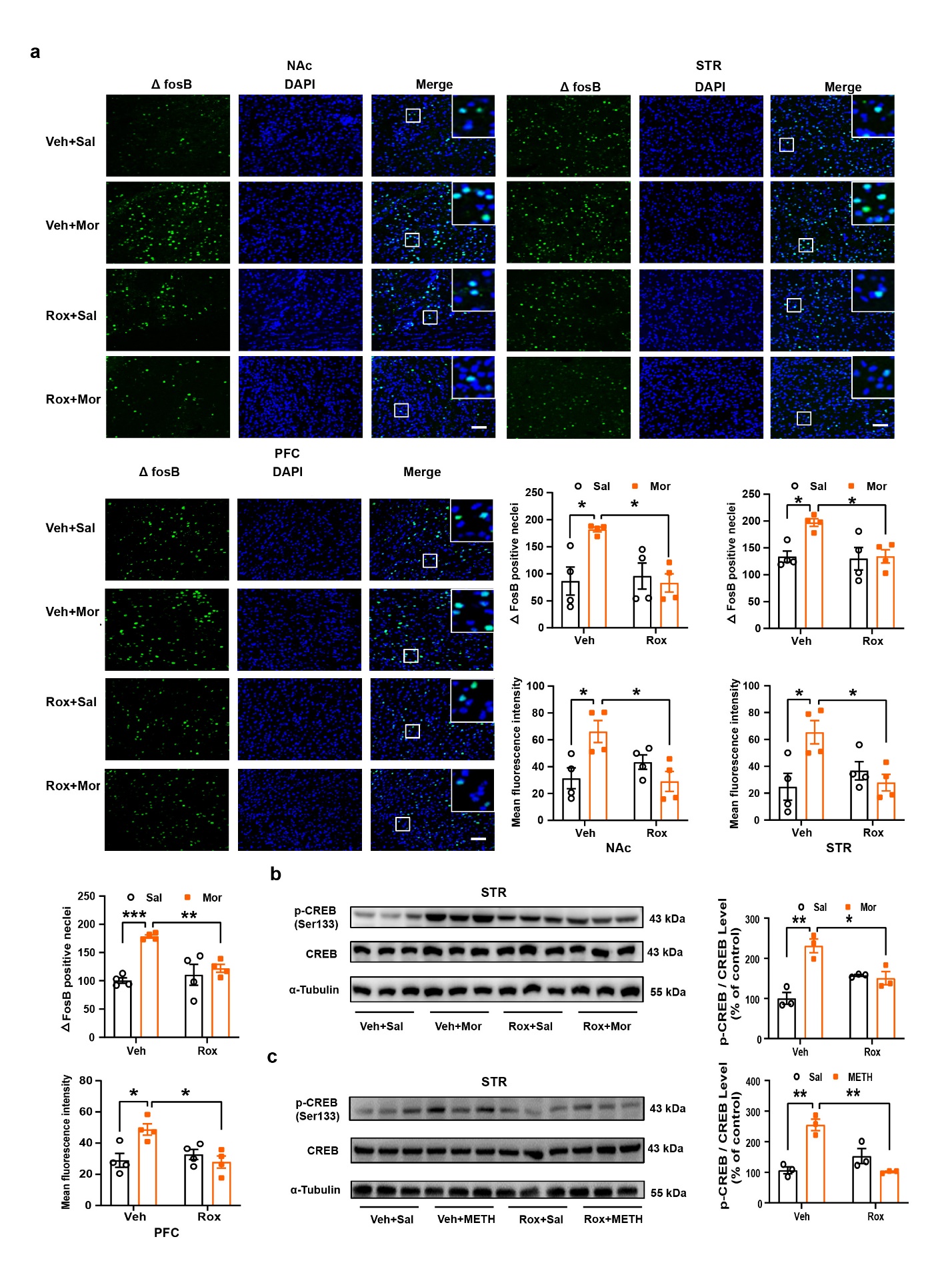
Figure. S2.

**Figure. S2. Rox inhibits morphine (Mor)-induced expression of Δ fosB and phosphorylated-CREB in mouse striatum.** Morphine-induced CPP was conducted as described, Rox (10 mg/kg) was administrated as indicated. Mouse brains were collected 24 hours after Mor-CPP test, and processed for immunofluorescence assay of Δ fosB or immunoblot of phosphorylated-CREB (p-CREB) using respective antibody. **(a)** Rox (10 mg/kg) inhibited Mor-induced expression of Δ FosB in striatum (STR: dorsomedial striatum), nucleus accumbens (NAc) shell and prefrontal cortex (PFC: anterior cingulate cortex) (Δ FosB positive nuclei: STR: *n* = 4, group, *F*_(1,12)_ = 6.131, *P* = 0.0292; drug, *F*_(1,12)_ = 5.951, *P* = 0.0312; group × drug, *F*_(1,12)_ = 4.689, *P* = 0.0512; NAc: *n* = 4, group, *F*_(1,12)_ = 4.349, *P* = 0.0580; drug, *F*_(1,12)_ = 5.165, *P* = 0.0422; group × drug, *F*_(1,12)_ = 7.513, *P* = 0.0179; PFC: *n* = 4, group, *F*_(1,12)_ = 19.00, *P* = 0.0009; drug, *F*_(1,12)_ = 5.129, *P* = 0.0428; group × drug, *F*_(1,12)_ = 10.49, *P* = 0.0071. Mean fluorescence intensity: STR: *n* = 4, group, *F*_(1,12)_ = 3.945, *P* = 0.0703; drug, *F*_(1,12)_ = 2.545, *P* = 0.1366; group × drug, *F*_(1,12)_ = 9.520, *P* = 0.0094; NAc: *n* = 4, group, *F*_(1,12)_ = 1.975, *P* = 0.1853; drug, *F*_(1,12)_ = 2.944, *P* = 0.1119; group × drug, *F*_(1,12)_ = 11.42, *P* = 0.0055; PFC: *n* = 4, group, *F*_(1,12)_ = 3.802, *P* = 0.0749; drug, *F*_(1,12)_ = 4.939, *P* = 0.0462; group × drug, *F*_(1,12)_ = 10.33, *P* = 0.0074). **(b-c)** Rox (10 mg/kg) inhibited Mor- or METH-induced expression of phosphorylated-CREB (S133) in mouse STR (**b**: *n* = 3, group, *F*_(1,8)_ = 19.62, *P* = 0.0022; drug, *F*_(1,8)_ = 0.6760, *P* = 0.4348; group × drug, *F*_(1,8)_ = 24.16, *P* = 0.0012. **c**: *n* = 3, group, *F*_(1,8)_ = 8.648, *P* = 0.0187; drug, *F*_(1,8)_ = 10.57, *P* = 0.0117; group × drug, *F*_(1,8)_ = 36.66, *P* = 0.0003). Representative images for immunofluorescence are shown in the left and upper panels and quantitative data are shown in the right and lower panels. Scale bar = 50 μmol/L. Representative images for immunoblots are shown in the left panels and quantitative data are shown in the right panels. Values are presented as means ± SEM. Statistical analyses were performed using two-way ANOVA followed by Bonferroni-corrected tests. **P* < 0.05, ***P* < 0.01, ****P* < 0.001. DAPI: 4',6-diamidino-2-phenylindole; Veh: vehicle; Sal: saline.


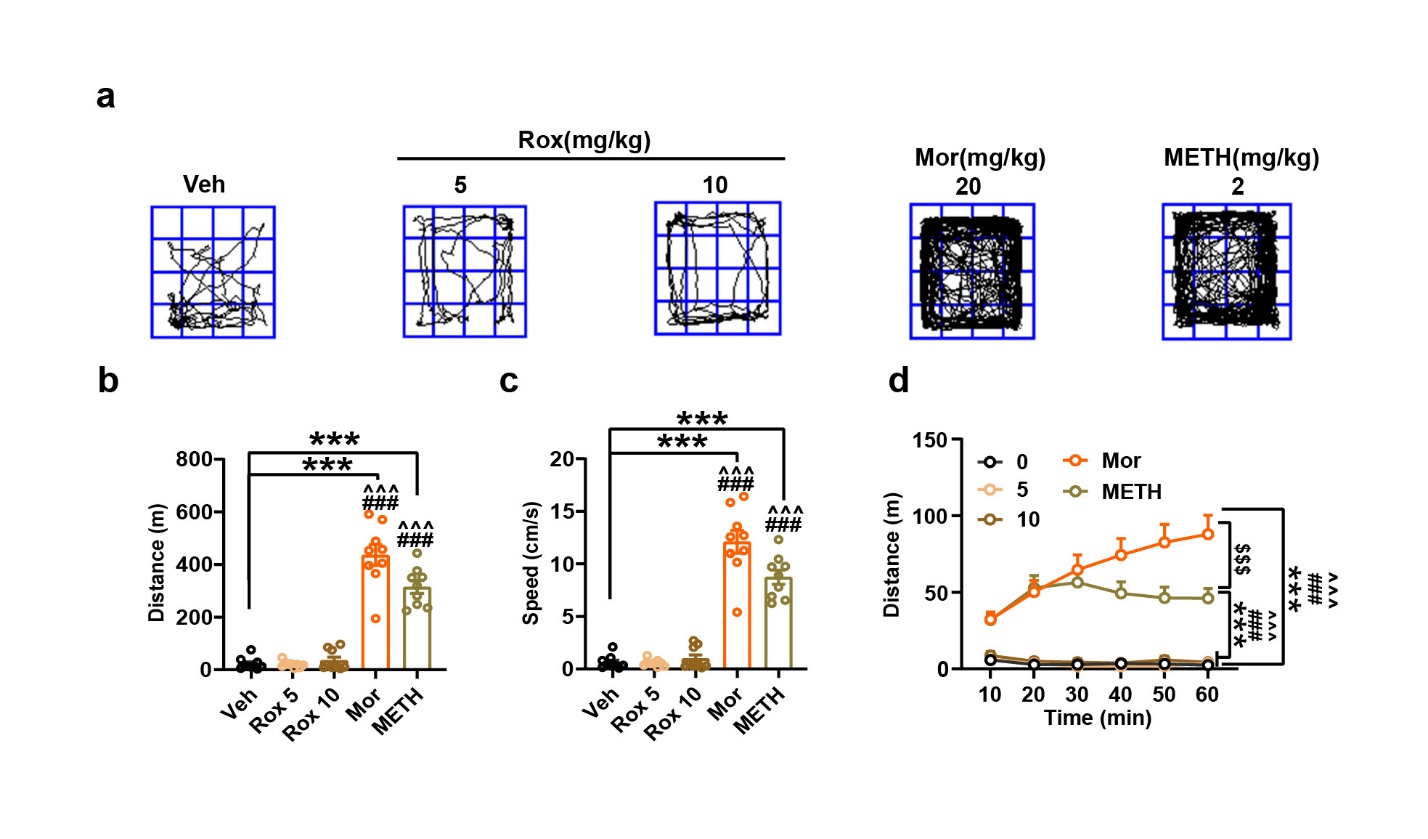
Figure. S3.

**Figure. S3. Rox treatment does not alter mouse locomotor activity.** Mice were treated with 0, 5, 10 mg/kg Rox 6 hours prior to behavioral tests. Locomotor activity was recorded 60 min after Rox, Mor and METH administration and analyzed. **(a)** mouse movement trajectory during 5 min; **(b)** total distance; **(c)** average speed; (**d)** distance traveled during each 10 min (*n* = 9, **b**, *F*_(4,40)_ = 80.63, *P* < 0.0001; **c**, *F*_(4,40)_ = 80.62, *P* < 0.0001; **d**, drug, *F*_(4,270)_ = 169.2, *P* < 0.0001, time, *F*_(5,270)_ = 3.129, *P* = 0.0092, drug × time, *F*_(20,270)_ = 3.464, *P* < 0.0001). Values are presented as means ± SEM. Statistical analyses for **b-c,** and for **d** were performed using one-way ANOVA and two-way ANOVA followed by Bonferroni-corrected tests, respectively. ****P* < 0.001; ^###^*P* < 0.001vs 5 mg/kg; ^^^*P* < 0.001vs 10 mg/kg; ^$$$^ *P* < 0.001 Mor vs METH.


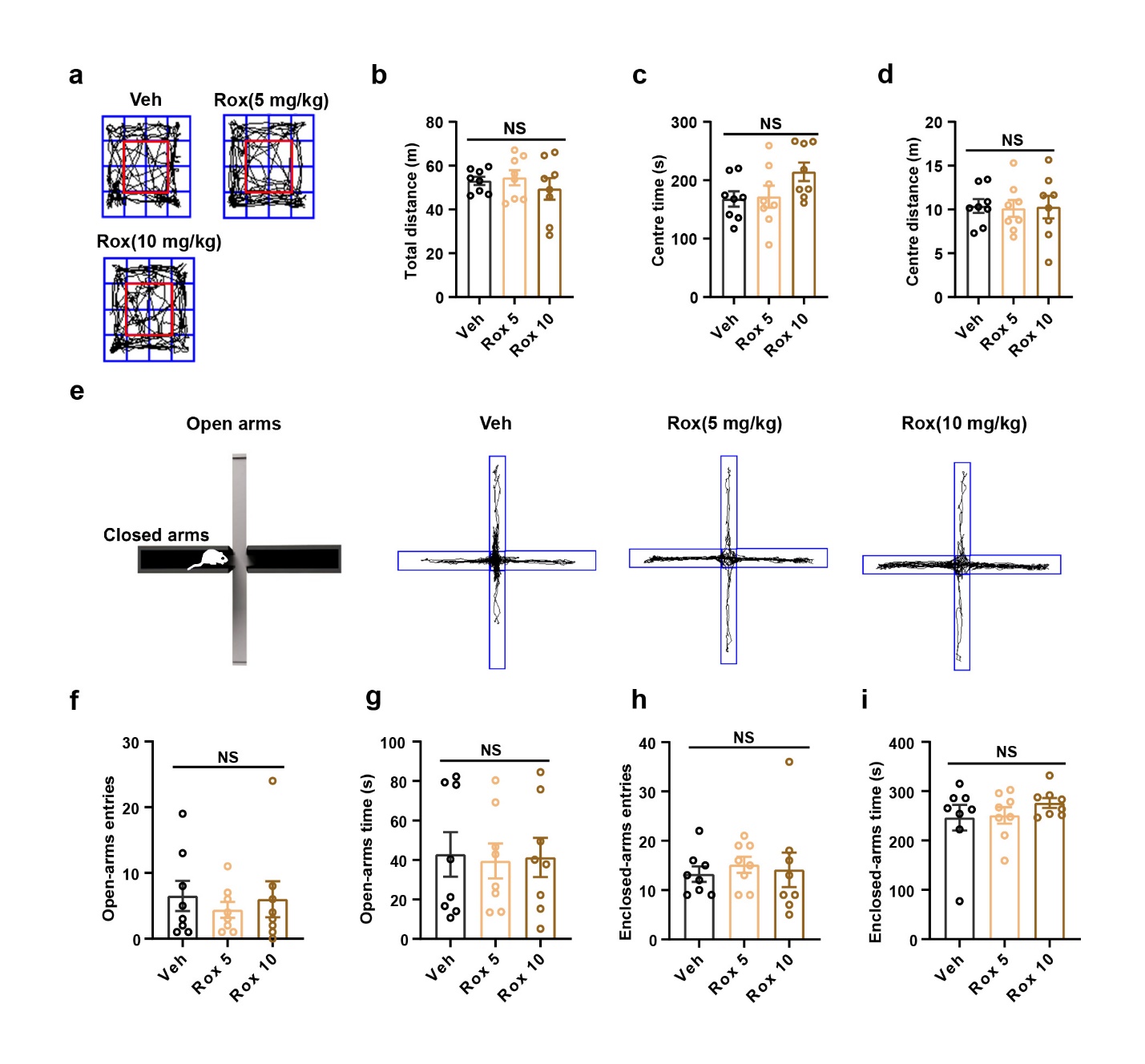
Figure. S4.

**Figure. S4. Rox treatment does not induce mouse anxiety-related behaviors.** Mice were treated with Rox (0, 5, 10 mg/kg) 6 hours prior to behavioral tests, locomotor activity was recorded 30 min and analyzed as follow: **(a)** mouse movement trajectory (30 min). **(b)** Rox treatment did not alter total distance (*n* = 8, *F*_(2,21)_ = 0.5168, *P* = 0.6038) or **(c-d)** centre time and centre distance (*n* = 8, **c**, *F*_(2,21)_ = 2.553, *P* = 0.1018; **d**, *F*_(2,21)_ = 0.01883, *P* = 0.9814). **(e)** mice movement trajectory in elevated plus maze (10 min recording). Rox did not induce anxiety as indicated by no alteration in **(f-g)** open-arms entries and time (*n* = 8, **f**, *F*_(2,21)_ = 0.2599, *P* = 0.7736; **g**, *F*_(2,21)_ = 0.02775, *P* = 0.9727); (**h-i**) enclosed-arms entries and time (*n* = 8, **h**, *F*_(2,21)_ = 0.1532, *P* = 0.8589; **i**, *F*_(2,21)_ = 0.7399, *P* = 0.4892). Values are presented as means ± SEM. Statistical analyses were performed using one-way ANOVA followed by Bonferroni-corrected tests. NS: not significant


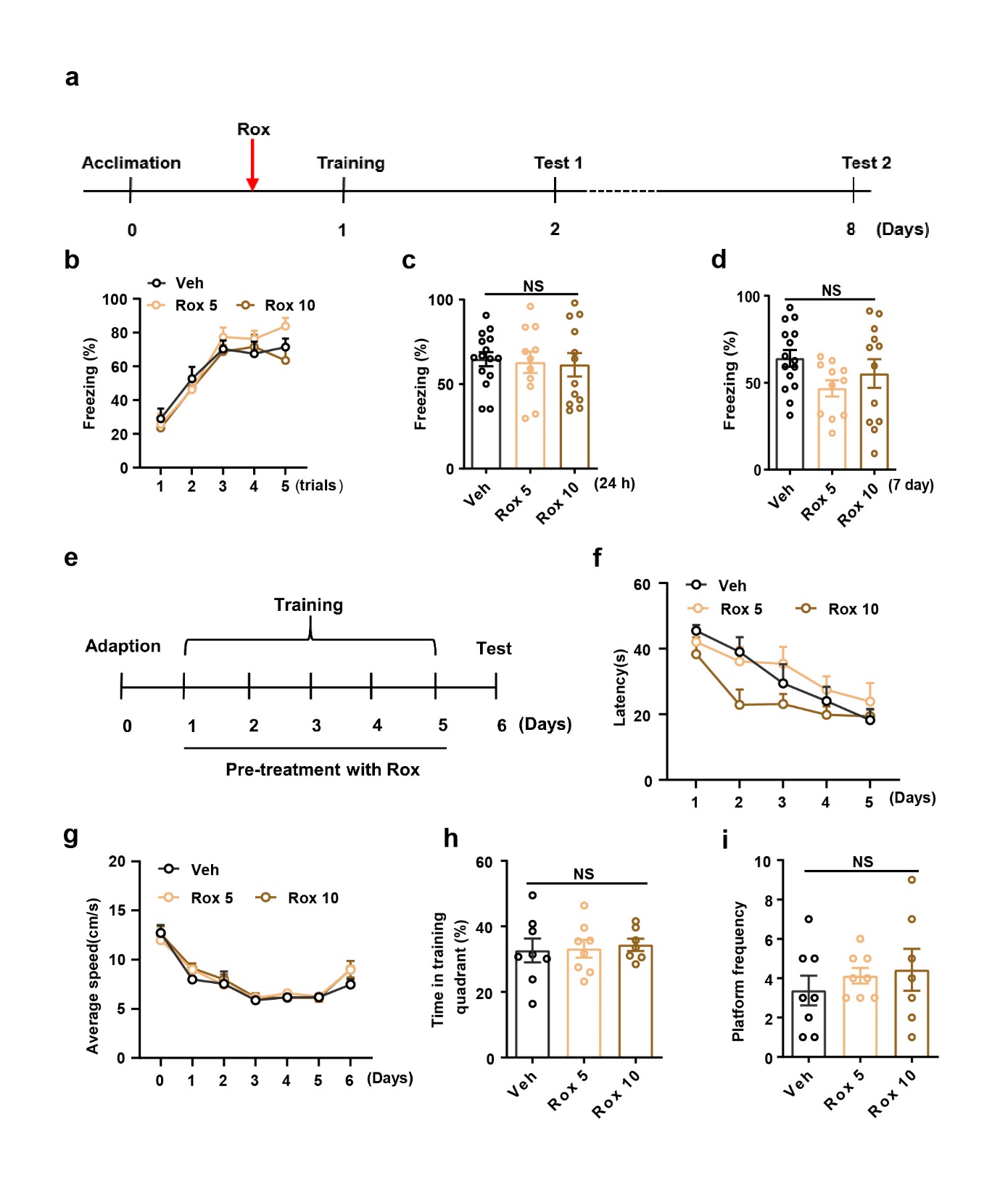
Figure. S5.

**Figure. S5. Rox treatment does not alter mouse fear conditioning memory or spatial memory.** Mice were treated with 0, 5, 10 mg/kg Rox 6 hours prior to training phase for fear conditioning and water maze. **(a)** Fear conditioning schematic diagram. **(b)** Learning curve; **(c)** 24 hours retrieval; (**d)** 7 days retrieval. Rox did not alter mouse fear acquisition and memory retrieval 24 hours and 7 days after training (**b**, Veh, *n* = 15, Rox 5, *n* = 12, Rox 10, *n* = 15, drug, *F*_(2,195)_ = 1.611, *P* = 0.2024, trials, *F*_(4,195)_ = 34.86, *P* < 0.0001, drug × trials, *F*_(8,195)_ = 0.6773, *P* = 0.7112. **c**, Veh, *n* = 15, Rox 5, *n* = 11, Rox 10, *n* = 12, *F*_(2,35)_ = 0.09250, *P* = 0.9119. **d**, Veh, *n* = 15, Rox 5, *n* = 11, Rox 10, *n* = 12, *F*_(2,35)_ = 2.048, *P* = 0.1442). **(e)** Water maze schematic diagram. **(f-i)** **f** Learning curve; **g** average speed; **h** time in training quadrant; **i** platform cross frequency. Rox had minimal effect on the mouse learning curve and did not alter swimming speed, time in training quadrant and platform crossing frequency in water maze (**f**, Veh, *n* = 8, Rox 5, *n* = 8, Rox 10, *n* = 7, drug, *F*_(2,100)_ = 5.158, *P* = 0.0074, trials, *F*_(4,100)_ = 11.80, *P* < 0.0001, drug × trials, *F*_(8,100)_ = 0.7439, *P* = 0.6526. **g,** Veh, *n* = 8, Rox 5, *n* = 8, Rox 10, *n* = 7, drug, *F*_(2,140)_ = 1.369, *P* = 0.2578, trials, *F*_(6,140)_ = 53.53, *P* < 0.0001, drug × trials, *F*_(12,140)_ = 0.6654, *P* = 0.7820. **h**, Veh, *n* = 8, Rox 5, *n* = 8, Rox 10, *n* = 7, *F*_(2,20)_ = 0.08837, *P* = 0.9158. **i**, Veh, *n* = 8, Rox 5, *n* = 8, Rox 10, *n* = 7, *F*_(2,35)_ = 0.5078, *P* = 0.6094). Values are presented as means ± SEM. Statistical analyses for **c-d** and **h-i,** and for **b, f** and **g** were performed using one-way ANOVA and two-way ANOVA followed by Bonferroni-corrected tests, respectively. NS: not significant.


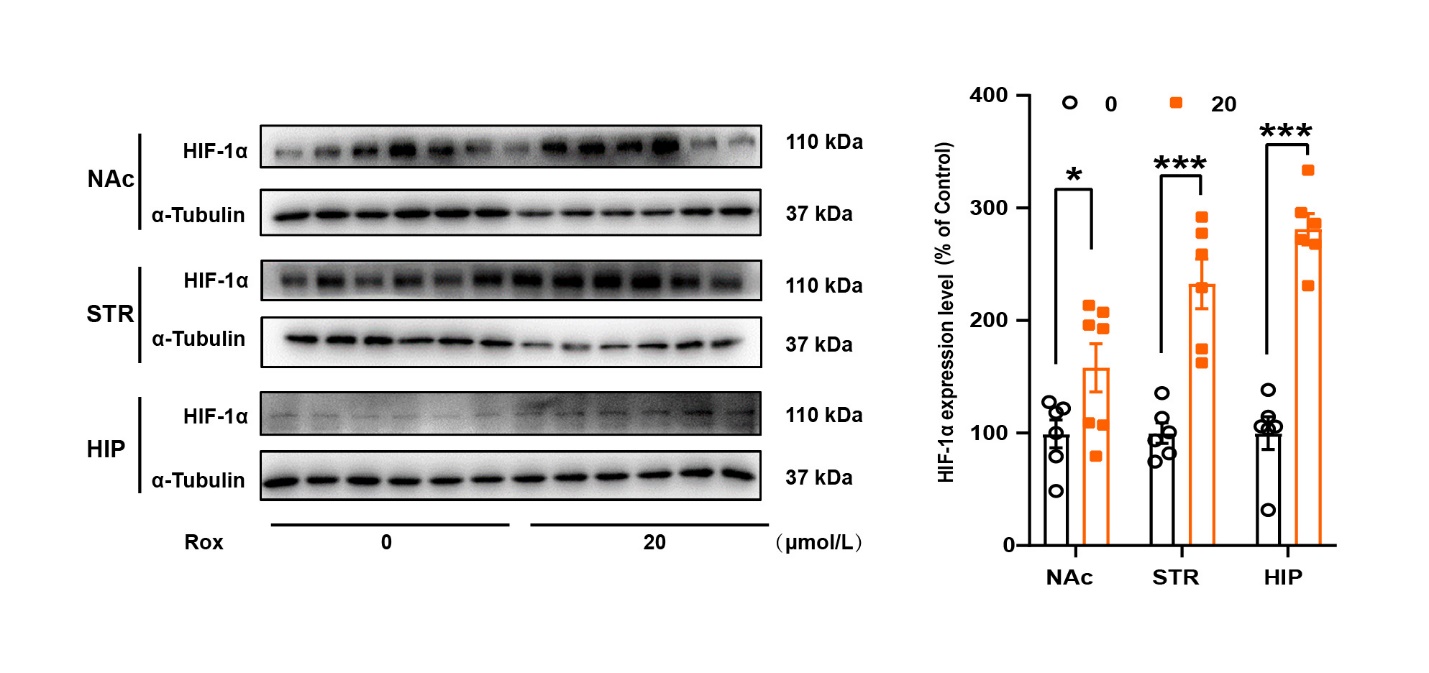
Figure. S6.

**Figure. S6. Local infusion of Rox increases HIF-1α expression in brain areas.** The bilateral cannula was implanted into WT mice brain in NAc, STR and HIP area, respectively. After 7 days interval, mice were received a single injection of 20 μmol/L roxadustat or vehicle for 2 hours. Then, the brain tissues were collected and prepared for immunoblot assays. A single injection of Rox increased HIF-1α expression in above regions (*n* = 7 for NAc-20 μmol/L Rox group*, n* = 6 for all other groups, drug, *F*_(1,31)_ = 82.79, *P* < 0.0001, area, *F*_(2,31)_ = 7.105, *P* = 0.0029, drug × area, *F*_(2,31)_ = 6.944, *P* = 0.0032). Representative images for immunoblots are shown in the left panels and quantitative data are shown in the right panels. Values are presented as means ± SEM. Statistical analyses were performed using two-way ANOVA followed by Bonferroni-corrected tests. **P* < 0.05, ****P* < 0.001.


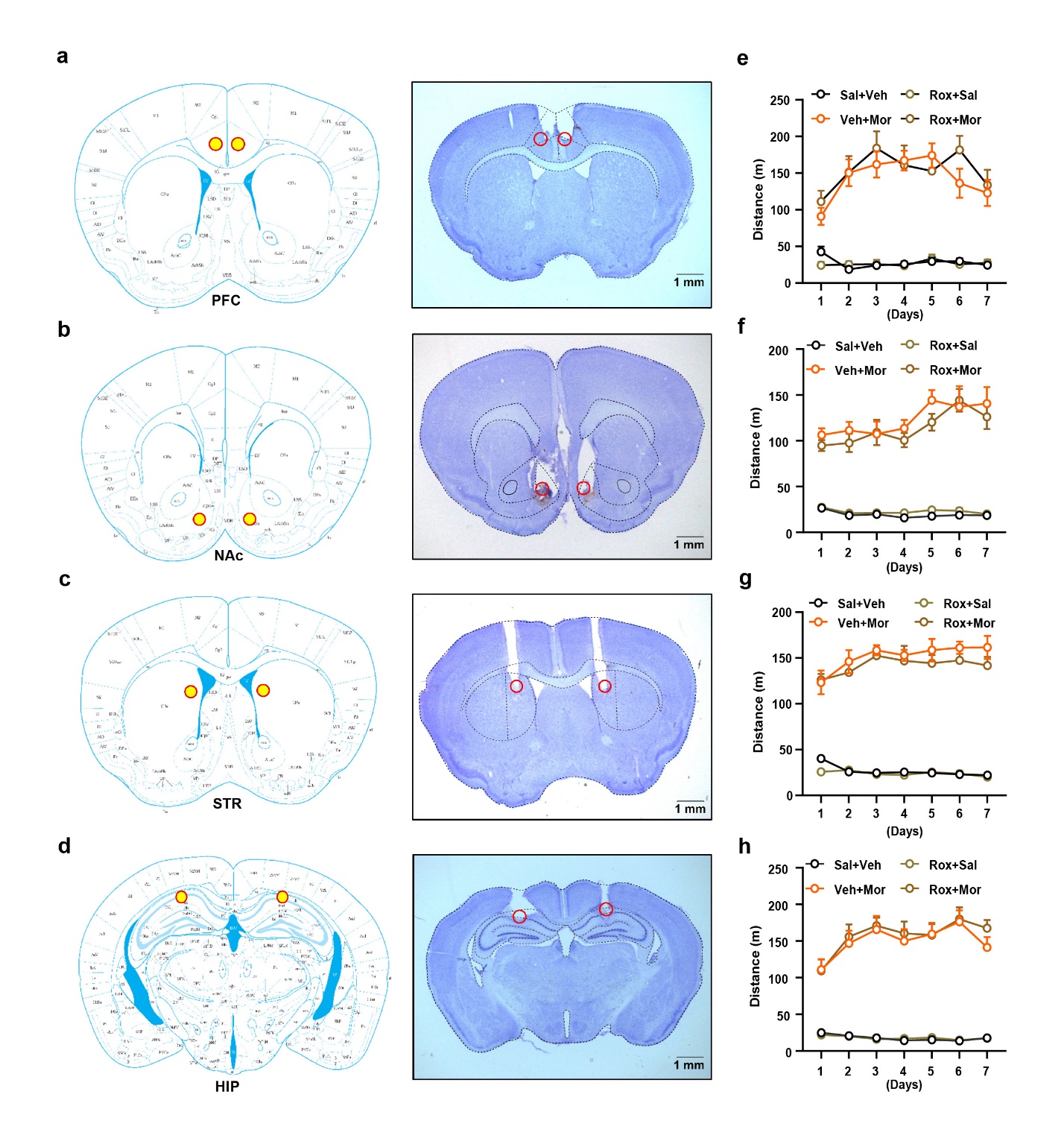
Figure. S7.

**Figure. S7. Location of bilateral cannula implantation and locomotor activity in Mor-CPP. (a-d)** Reference position of bilateral cannula implantation area and Nissl staining of mouse brain with Rox administration into respective PFC, NAc, STR and HIP. At least 3 mice were used for Nissl staining in each group. **(e-h)** The locomotor activity in CPP training of bilateral cannula implanted mice after morphine or saline administration. Rox or DFP alone did not alter mouse locomotor activity after morphine or saline administration (**e**, Sal+Veh, *n* = 10, Veh+Mor, *n* = 15, Rox+Sal, *n* = 9, Rox+Mor, *n* = 15; **f**, Sal+Veh, *n* = 12, Veh +Mor, *n* = 15, Rox+Sal, *n* = 12, Rox+Mor, *n* = 14; **g**, Sal+ Veh, *n* = 10, Veh+Mor, *n* = 12, Rox+Sal, *n* = 10, Rox+Mor, *n* = 12; **h**, Sal+ Veh, *n* = 12, Veh +Mor, *n* = 14, Rox+Sal, *n* = 11, Rox+Mor, *n* = 14). Values are presented as means ± SEM. Statistical analyses were performed using two-way ANOVA followed by Bonferroni-corrected tests.


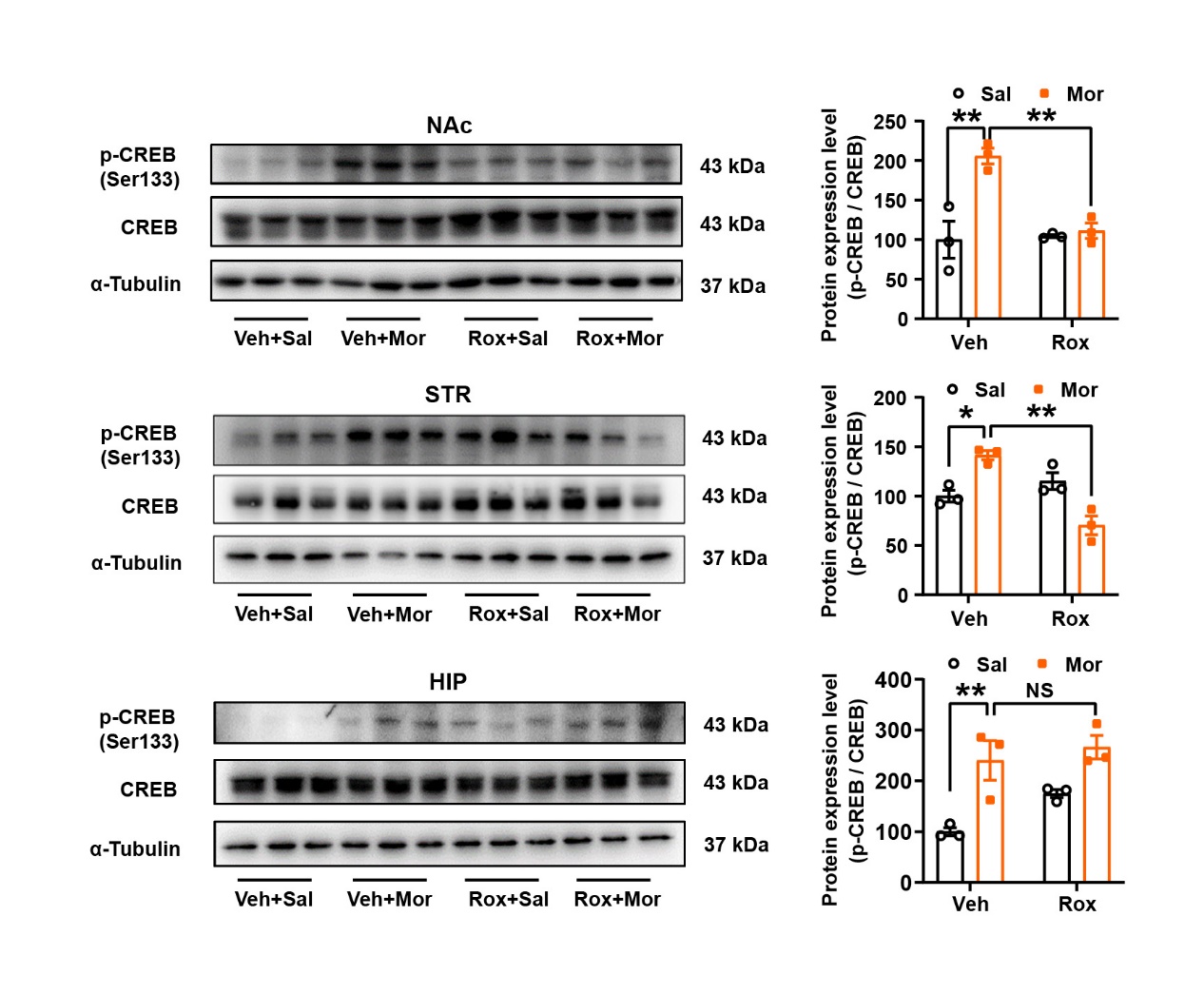
Figure. S8.

**Figure. S8. Local infusion of Rox inhibits Mor-induced expression of phosphorylated-CREB (p-CREB).** The bilateral cannula was implanted into NAc, STR and HIP areas of of WT mice. After 7 days interval, the mice were used to perform CPP experiment as described in Methods. The brain tissues were then collected 24 hours after Mor-CPP test and prepared for immunoblot assays of p-CREB. Rox pre-treatment suppressed the Mor-induced expression of (Ser133) p-CREB in NAc and STR, but not in HIP (NAc, *n* = 3 for each group, group, *F*_(1,8)_ = 16.95, *P* = 0.0034, drug, *F*_(1,8)_ = 10.85, *P* = 0.0110, drug × area, *F*_(1,8)_ = 13.14, *P* = 0.0067. STR, *n* = 3 for each group, group, *F*_(1,8)_ = 0.05597, *P* = 0.8189, drug, *F*_(1,8)_ = 14.12, *P* = 0.0056, drug × area, *F*_(1,8)_ = 33.71, *P* = 0.0004. HIP, *n* = 3 for each group, group, *F*_(1,8)_ = 24.51, *P* = 0.0011, drug, *F*_(1,8)_ = 4.701, *P* = 0.0620, drug × area, *F*_(1,8)_ = 1.096, *P* = 0.3257). Representative images for immunoblots are shown in the left panels and quantitative data are shown in the right panels. Values are presented as means ± SEM. Statistical analyses were performed using two-way ANOVA followed by Bonferroni-corrected tests. **P* < 0.05, ***P* < 0.01. NS: not significant.


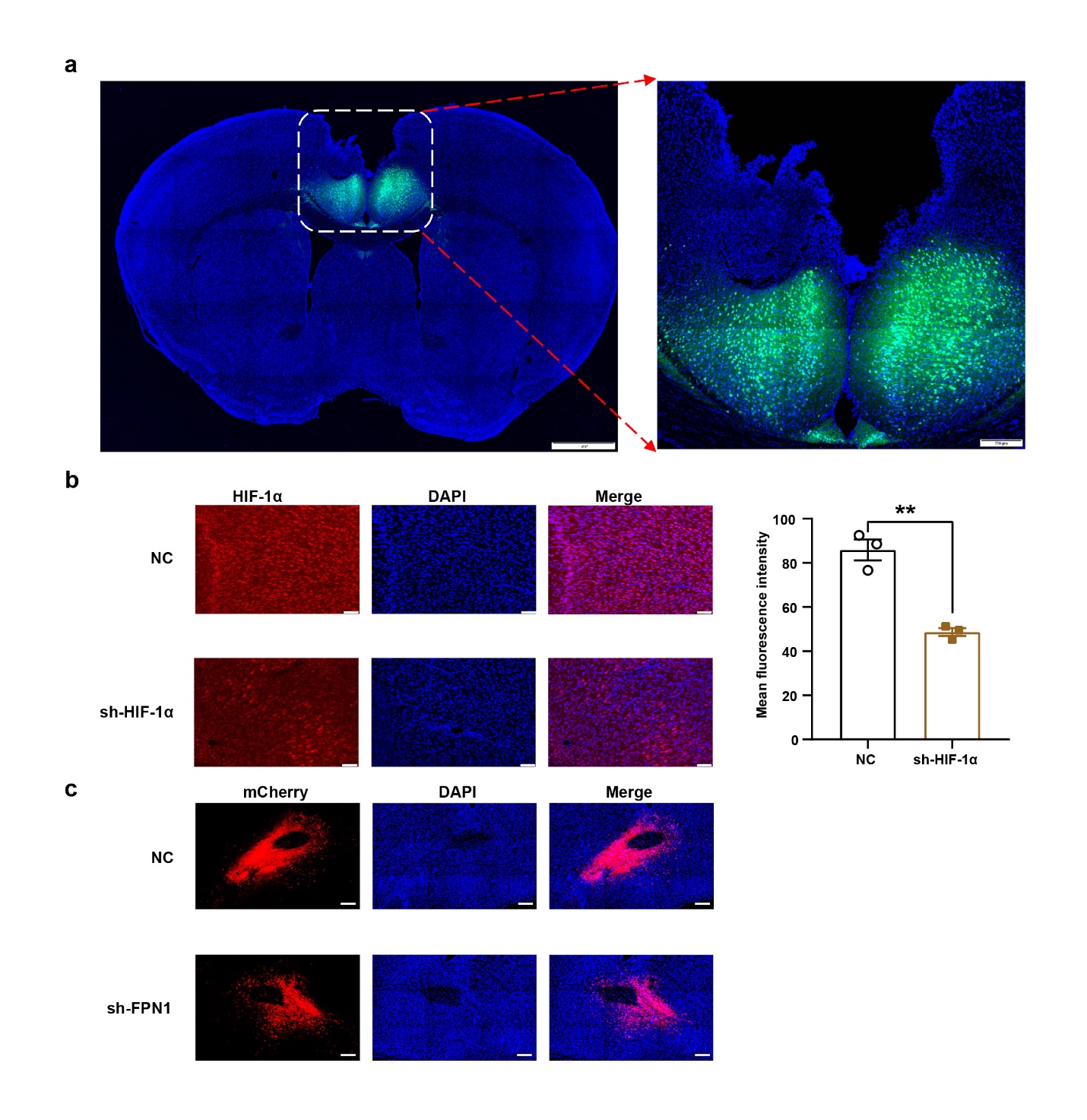
Figure. S9.

**Figure. S9. Verification of knockdown efficiency of neuronal specific AAV-shHIF-1α.** Mouse brain was collected 21 days after AAV injection. **(a-b)** Injection location and knockdown efficiency of HIF-1α was confirmed as indicated by reduction in mean fluorescence intensity relative to NC in PFC (*n* = 3, *t* **=** 7.318, *df* = 4, *P* = 0.0019). **(c)** The AAV expression in mouse NAc after 21 days injection (red: mCherry). Representative images for immunofluorescence are shown in the left panels and quantitative data are shown in the right panels. Scale bar = 50 μmol/L.Scar bar: **a**, 1 mm; **b** and **c**, 200 μmol/L. Values are presented as means ± SEM. Statistical analysis was performed using Student’s *t*-test. ***P* < 0.01.


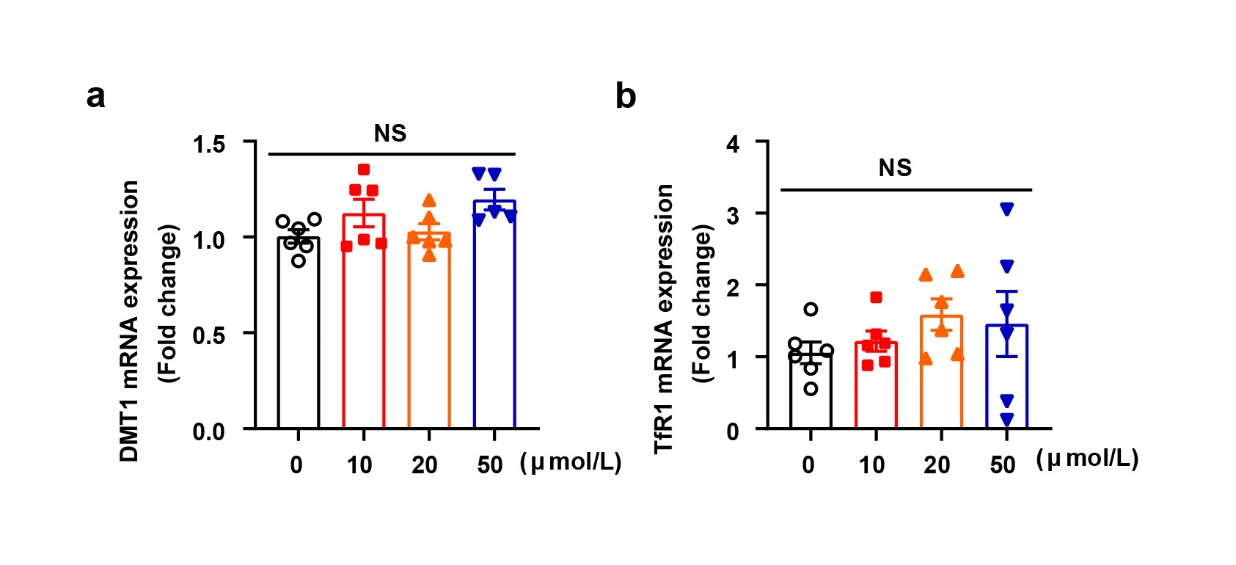
Figure. S10.

**Figure. S10. Rox treatment does not alter iron import gene expression.** (**a-b**) PC12 cells were treated with Rox for 24 hours at indicated concentration. Then, the cells were collected and processed for q-PCR assays to detect Rox treatment-altered mRNA expression of divalent metal-ion transporter-1 (DMT1) and transferrin receptor 1 (TfR1) in PC12 cells (**a**: 0,10,20, 50 μmol/L, *n* = 6) *n* = 5-6, *F*_(3,19)_ = 2.724, *P* = 0.0729; **b**: *n* = 6, *F*_(3,20)_ = 0.7671, *P* = 0.5259). Values are presented as means ± SEM. Statistical analyses were performed using one-way ANOVA followed by Bonferroni-corrected tests. NS: not significant.


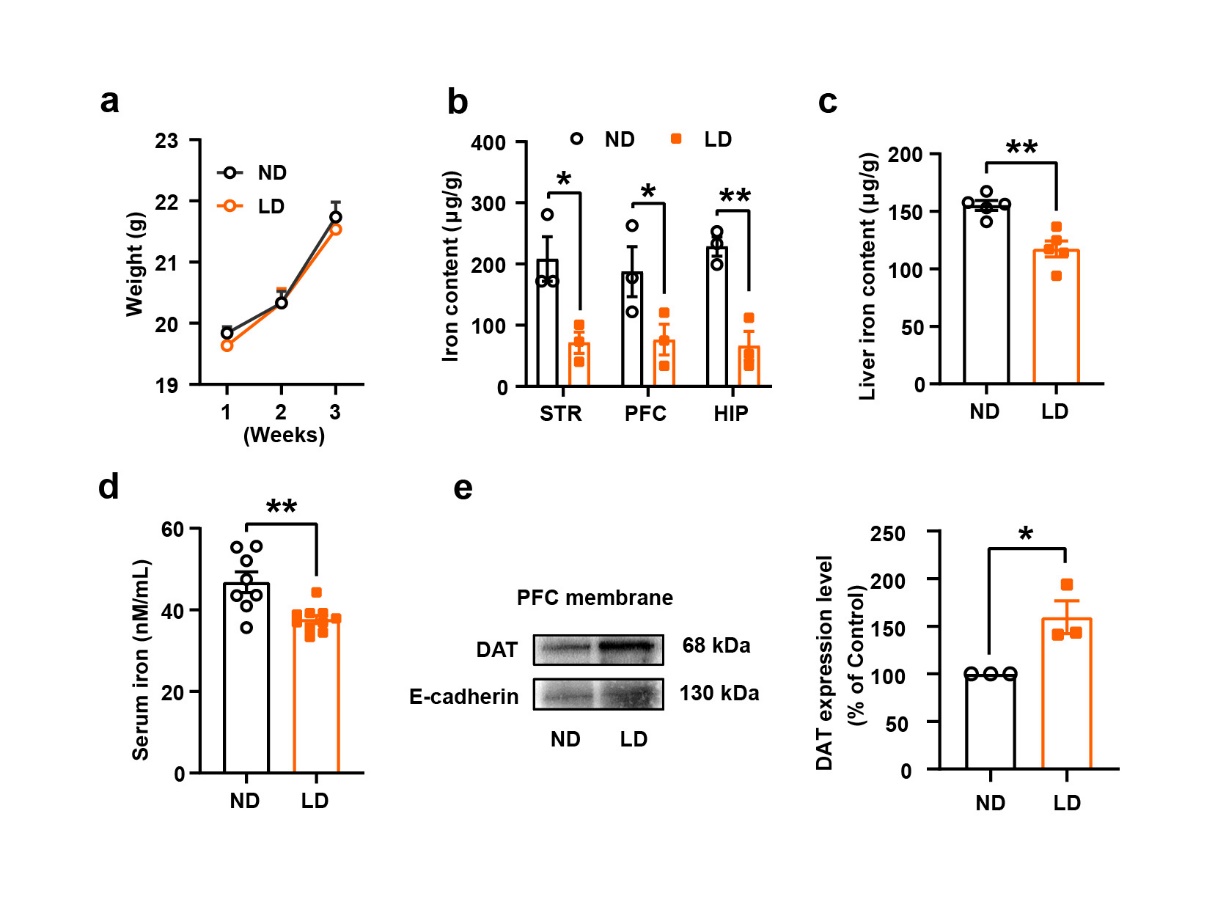
Figure. S11.

**Figure. S11. Low iron diet induces iron deficiency and plasma membrane DAT upregulation without altering body weight.** Mice were fed with low iron diet for 4 weeks as described in Methods. Low iron diet (LD) did not (**a**) change body weight. (normal iron diet,ND) (normal iron diet,ND;  *n* = 15, diet, *F*_(1,84)_ = 0.7151, *P* = 0.4001, week, *F*_(2,84)_ = 48.81, *P* < 0.0001, diet × week, *F*_(2,84)_ = 0.1789, *P* = 0.8365). **(b-d)** The iron content of the STR, PFC, HIP, liver, and serum were analyzed by using ICP-MS was significantly decreased (**b**, STR, PFC and HIP, *n* = 3, diet, *F*_(1,12)_ = 35.35, *P <* 0.0001; area, *F*_(2,12)_ = 0.1528, *P* = 0.8599; diet × area, *F*_(2,12)_ = 0.4119, *P* = 0.6714. **c**, liver, *n* = 5, *t* = 4.602, *df* = 8, *P =* 0.0018; **d**, serum, ND, *n* = 8, LD, *n* = 10, *t* = 3.654, *df* = 16, *P =* 0.0021). **(e)** Mouse brain tissues (PFC) were collected after 4 weeks low iron diet for the detection of membrane DAT (*n* = 3, *t* = 3.462, *df* = 4, *P =* 0.0258). Representative images for immunoblots are shown in the left panels and quantitative data are shown in the right panels. Values are presented as means ± SEM. Statistical analyses for **a-b**, and for **c-e** were performed using two-way ANOVA followed by Bonferroni-corrected tests and Student’s *t*-test, respectively. **P* < 0.05, ***P* < 0.01. ND: normal iron diet; LD: low iron diet.


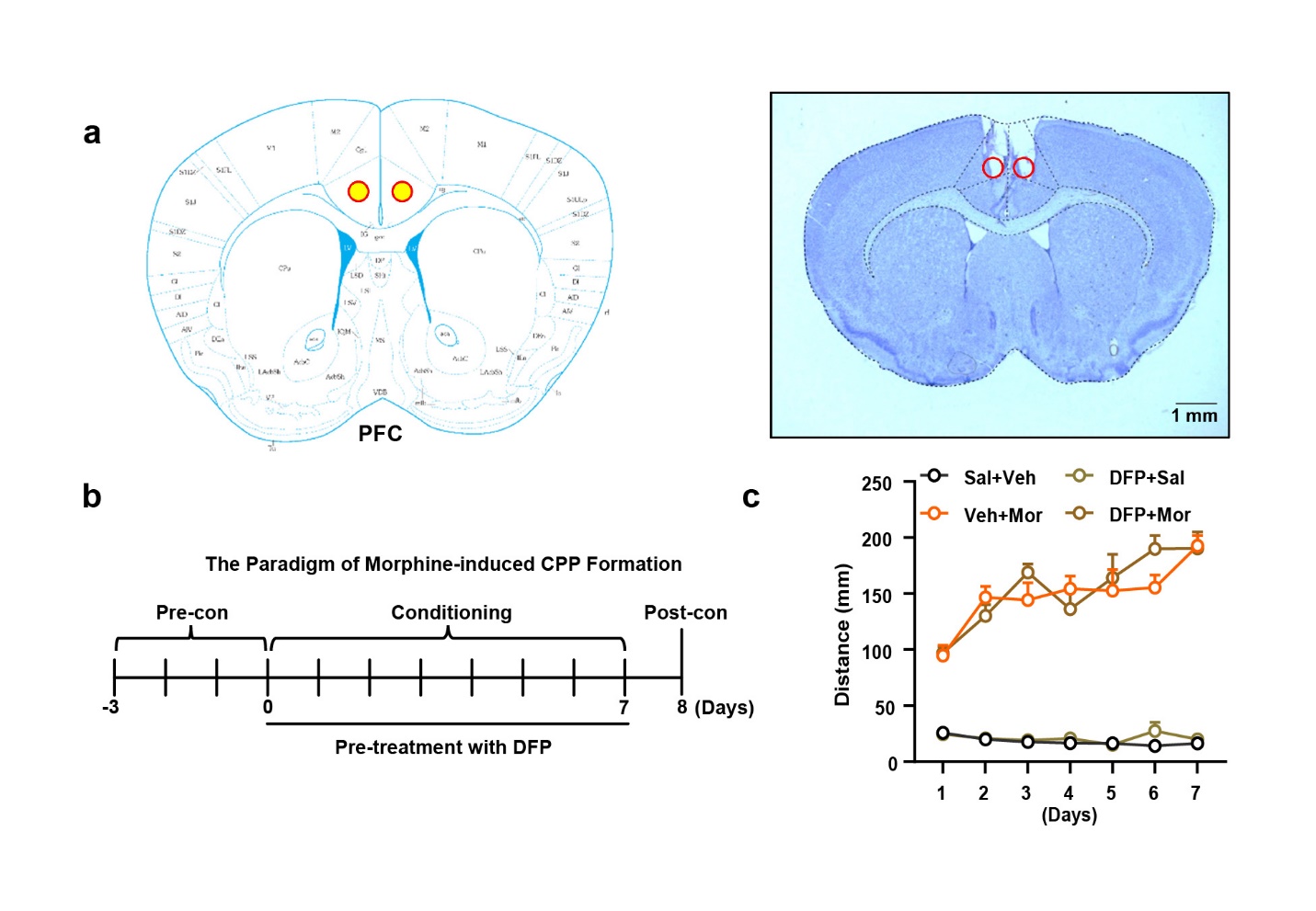
Figure. S12.

**Figure. S12. Location of bilateral cannula implantation and locomotor activity in Mor-CPP. (a)** Reference position of bilateral cannula implantation area and Nissl staining of mouse brain for deferiprone (DFP) administration into PFC. At least 3 mice were used for Nissl staining in each group. **(b)** The paradigm of Mor-CPP formation and DFP treatment (2 μL per site, 13 mg/mL 2 hours before morphine injection in the conditioning phase. **(c)** The locomotor activity in CPP training of bilateral cannula implanted mice after morphine or saline administration. DFP alone did not alter mouse locomotor activity after morphine or saline administration (Sal+Veh, *n* = 10, Veh +Mor, *n* = 13, Rox+Sal, *n* = 10, Rox+Mor, *n* = 13). Values are presented as means ± SEM. Statistical analyses were performed using two-way ANOVA followed by Bonferroni-corrected tests.


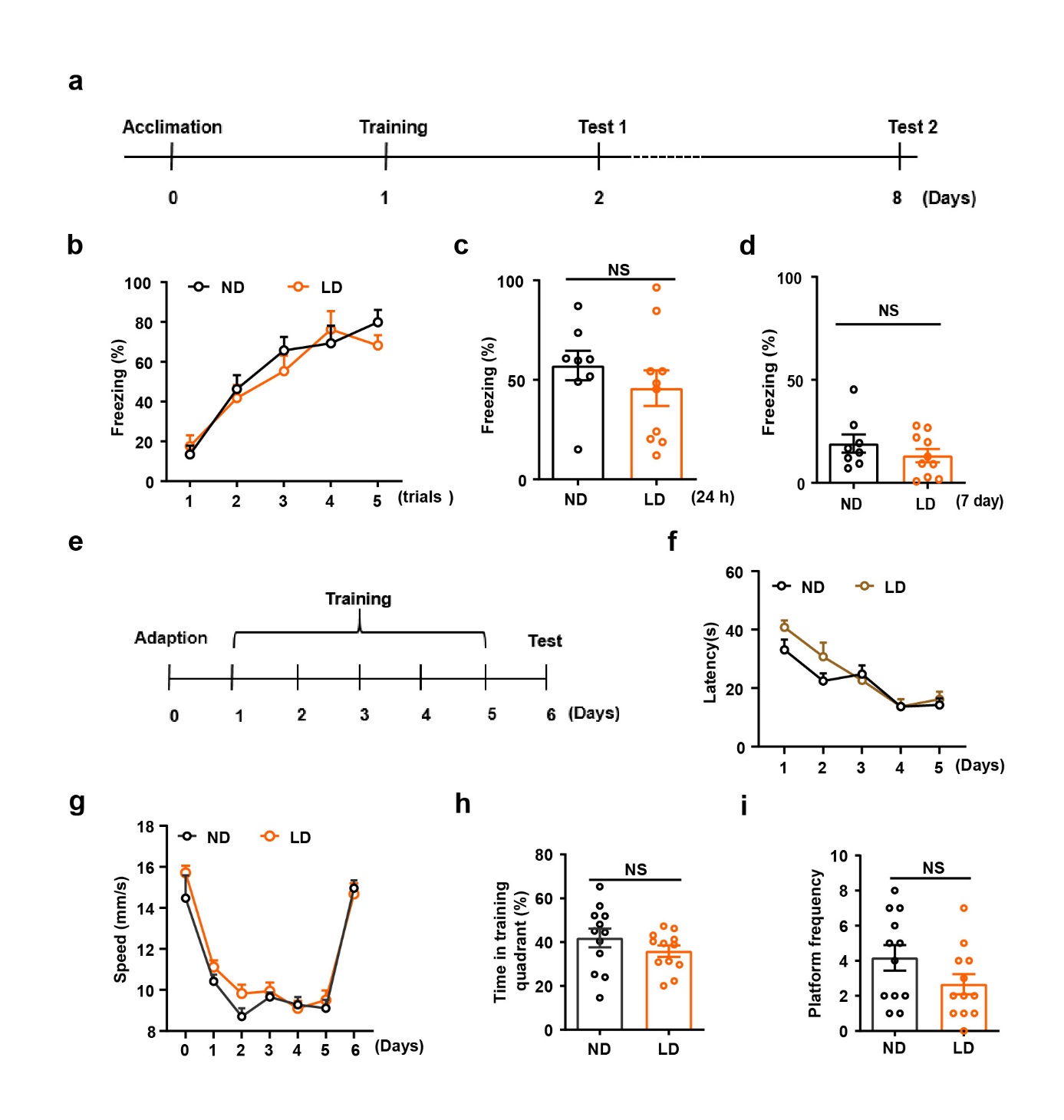
Figure. S13.

**Figure. S13. Iron deficiency does not alter mouse fear conditioning memory or spatial memory.** Mice were fed with low iron diet for 4 weeks as described in Methods. Then, mice were subjected to test for fear conditioning and water maze performance. **(a)** Fear conditioning schematic diagram. **(b)** Learning curve; **(c)** 24 hours retrieval; (**d)** 7 days retrieval. Low iron diet did not alter mouse fear acquisition and memory retrieval 24 hours and 7 days after training (**b**, ND, *n* = 8, LD, *n* = 10, diet, *F*_(1,80)_ = 0.4785, *P* = 0.4911, trials, *F*_(4,80)_ = 24.04, *P* < 0.0001, diet × trials, *F*_(4,80)_ = 0.7125, *P* = 0.5858. **c**, ND, *n* = 8, LD, *n* = 10, *t* = 0.9389, *df* = 16, *P* = 0.3617. **d**, ND, *n* = 8, LD, *n* = 10, *t* = 1.094, *df* = 16, *P* = 0.2903). **(e)** Water maze schematic diagram. **(f)** Learning curve; **(g)** average speed; (**h**) time in training quadrant; (**i**) platform cross frequency. Low iron diet did not alter mouse learning curve, swimming speed, time in training quadrant and platform crossing frequency in water maze (**f**, ND, *n* = 12, LD, *n* = 12, diet, *F*_(1,110)_ = 3.095, *P* = 0.0813, trials, *F*_(4,110)_ = 21.78, *P* < 0.0001, diet × trials, *F*_(4,110)_ = 1.333, *P* = 0.2621. **g**, ND, *n* = 12, LD, *n* = 12, diet, *F*_(1,154)_ = 3.327, *P* = 0.0701, trials, *F*_(6,154)_ = 60.60, *P* < 0.0001, diet × trials, *F*_(6,154)_ = 0.7546, *P* = 0.6067. **h**, ND, *n* = 12, LD, *n* = 12, *t* = 1.200, *df* = 22, *P* = 0.2427. **i**, ND, *n* = 12, LD, *n* = 12, *t* = 1.612, *df* = 22, *P* = 0.1213). Values are presented as means ± SEM. Statistical analyses for **b, f** and **g**, and for **c-d** and **h-i** were performed using two-way ANOVA followed by Bonferroni-corrected tests and Student’s *t-*test, respectively. NS: not significant.

Table S1.

**Table S1** Blood routine examination

| Weeks | Type | RBC (10^^12^/L) | HB (g/L) | HCT (%) |
| --- | --- | --- | --- | --- |
| 1 | ND | 7.08±0.07 | 112.00±1.88 | 35.40±0.69 |
|  | LD | 7.53±0.10 | 117.00±2.06 | 39.53±0.58 |
| 2 | ND | 8.71±0.09 | 134.25±1.24 | 43.28±0.33 |
|  | LD | 7.77±0.14 | 116.50±2.16 | 37.53±0.64 |
| 3 | ND | 8.69±0.03 | 131.00±0.54 | 43.15±0.14 |
|  | LD | 8.48±0.13 | 126.80±2.31 | 41.00±0.60 |
| 4 | ND | 8.00±0.08 | 117.67±0.96 | 40.40±0.45 |
|  | LD | 9.11±0.08 | 132.25±2.17 | 46.43±0.38 |
| 5 | ND | 8.06±0.15 | 111.67±2.45 | 40.37±0.73 |
|  | LD | 8.18±0.10 | 108.33±1.48 | 39.20±0.61 |
|  | Reference values | 6.36-9.42 | 110.00-143.00 | 34.60-44.60 |

**Table S1. Low iron diet induced iron deficiency without altering routine blood tests.** Mice were fed with low iron diet as described in Methods. The blood routine examination was performed every week in experimental period. Low iron diet did not affect anemia (*n* = 4 for each group in week1; *n* = 4 for each group in week2; ND, *n* = 4, LD, *n* = 5 in week3; ND, *n* = 3, LD, *n* = 4 in week4; *n* = 3 for each group in week5) in mice. ND: normal iron diet; LD: low iron diet.

Table S2.

**Table S2** Sequences of the primers used for RT-PCR

| Species | Gene | Primer | Sequence |
| --- | --- | --- | --- |
| Rat | *Hepcidin* | Forward | 5'-GAAGGCAAGATGGCACTAAGCA-3' |
|  |  | Reverse | 5'-TCTCGTCTGTTGCCGGAGATAG-3' |
| Rat | *TfR1* | Forward | 5'-CGAAGTCCAGTGTGGGAACA-3' |
|  |  | Reverse | 5'-TGGCACCAACAGCTCCATAG-3' |
| Rat | *DMT1* | Forward | 5'-GGCAGTGTTTGATTGCGTTG-3' |
|  |  | Reverse | 5'-TAGTATTGCCACCGCTGGTATC-3' |
| Rat | *HIF-1α* | Forward | 5'-TGAAGAAGAATTAAACCCAA-3' |
|  |  | Reverse | 5'-AGTAACGTTCCAATTCCT-3' |
| Rat | *VEGF* | Forward | 5'-AACCATGAACTTTCTGCTCTC-3' |
|  |  | Reverse | 5'-CTTCATGGGCTTTCTGCTCC-3' |
| Rat | *β-actin* | Forward | 5'-CCCTAAGGCCAACCGTGAAAAG-3' |
|  |  | Reverse | 5'-TACGTACATGGCTGGGGTGT-3' |
| Human | *DAT* | Forward | 5'-CCACCATGCCATACGTGGTC-3' |
|  |  | Reverse | 5'-CCGGTAGAAGTCAACGCTCA-3' |
| Human | *GAPDH* | Forward | 5'-TCCAAAATCAAGTGGGGCGA-3' |
|  |  | Reverse | 5'-AAATGAGCCCCAGCCTTCTC-3' |
